# Differentially Tolerized Mouse Antigen Presenting Cells Share a Common miRNA Signature Including Enhanced mmu-miR-223-3p Expression Which Is Sufficient to Imprint a Protolerogenic State

**DOI:** 10.3389/fphar.2018.00915

**Published:** 2018-08-17

**Authors:** Matthias Bros, Mahmoud Youns, Verena Kollek, Diana Buchmüller, Franziska Bollmann, Ean-Jeong Seo, Jonathan Schupp, Evelyn Montermann, Svetlana Usanova, Hartmut Kleinert, Thomas Efferth, Angelika B. Reske-Kunz

**Affiliations:** ^1^Department of Dermatology, University Medical Center, Johannes Gutenberg University, Mainz, Germany; ^2^Department of Biochemistry and Molecular Biology, Faculty of Pharmacy, Helwan University, Cairo, Egypt; ^3^Department of Pharmacology, University Medical Center, Johannes Gutenberg University, Mainz, Germany

**Keywords:** dexamethasone, interleukin-10, miRNA, mmu-miR-223-3p, tolerogenic dendritic cells

## Abstract

Dendritic cells (DCs) are pivotal for the induction and maintenance of antigen-specific tolerance and immunity. miRNAs mediate post-transcriptional gene regulation and control in part the differentiation and stimulation-induced immunogenic function of DCs. However, the relevance of miRNAs for the induction and maintenance of a tolerogenic state of DCs has scarcely been highlighted yet. We differentiated mouse bone marrow cells to conventional/myeloid DCs or to tolerogenic antigen presenting cells (APCs) by using a glucocorticoid (dexamethasone) or interleukin-10, and assessed the miRNA expression patterns of unstimulated and LPS-stimulated cell populations by array analysis and QPCR. Differentially tolerized mouse APCs convergingly down-regulated a set of miRNA species at either state of activation as compared with the corresponding control DC population (mmu-miR-9-5p, mmu-miR-9-3p, mmu-miR-155-5p). These miRNAs were also upregulated in control DCs in response to stimulation. In contrast, miRNAs that were convergingly upregulated in both tolerized APC groups at stimulated state (mmu-miR-223-3p, mmu-miR-1224-5p) were downregulated in control DCs in response to stimulation. Overexpression of mmu-miR-223-3p in DCs was sufficient to prevent stimulation-associated acquisition of potent T cell stimulatory capacity. Overexpression of mmu-miR-223-3p in a DC line resulted in attenuated expression of known (Cflar, Rasa1, Ras) mRNA targets of this miRNA species shown to affect pathways that control DC activation. Taken together, we identified sets of miRNAs convergingly regulated in differentially tolerized APCs, which may contribute to imprint stimulation-resistant tolerogenic function as demonstrated for mmu-miR-223-3p. Knowledge of miRNAs with protolerogenic function enables immunotherapeutic approaches aimed to modulate immune responses by regulating miRNA expression.

## Introduction

In their activated state, dendritic cells (DCs) represent highly potent antigen presenting cells (APCs), which are capable to stimulate even naive T cells (Devi and Anandasabapathy, 2017). Stimuli that induce maturation comprise proinflammatory cytokines (Sozzani et al., 2017), pathogen-associated molecular patterns (Kowalski and Li, 2017) and endogenous danger-associated molecules like heat shock proteins (Nefla et al., 2016). Activated DCs express MHC and costimulatory molecules at high levels and secrete proinflammmatory cytokines and T cell-attracting chemokines (Joffre et al., 2009). Thereby, DCs induce differentiation of naïve antigen-specific T cells into T effector cells of various polarization.

In contrast, immature, and semi-mature DCs induce tolerance (Iberg et al., 2017). A tolerogenic state of DCs can be achieved *in vitro* by treatment of DC progenitors or immature DCs with anti-inflammatory cytokines like IL-10 or TGF-ß or with immunosuppressive agents like glucocorticoids and vitamin D3 metabolites (Kushwah and Hu, 2011). Such mediators inhibit the differentiation and/or maturation of DCs despite the presence of activating stimuli. In addition, they also facilitate upregulation of inhibitory cell surface molecules and the production of anti-inflammatory cytokines (Trojandt et al., 2016). Tolerogenic DCs induce T cell apoptosis, T cell anergy or the differentiation of regulatory T cells (Iberg et al., 2017). Due to their immune-modulatory potential, DCs are in the focus of developing immunotherapeutic approaches to combat various diseases like cancer, autoimmunity or allergies (Constantino et al., 2017).

A number of studies has highlighted the overall importance of non-coding short RNAs of about 19–24 nucleotides in length, termed microRNAs (miRNAs) (Kobayashi and Tomari, 2016) for immune cell differentiation and functions (Forero et al., 2017). These post-transcriptional regulators bind to evolutionarily conserved, (im)perfect complementary sequence stretches of target mRNAs located most often within the untranslated regions. Binding of a miRNA to a target mRNA mostly results either in an enhanced mRNA decay or in an attenuated mRNA translation rate as facilitated by recruitment of different nucleases and interference with the ribosomal translation machinery, respectively (Iwakawa and Tomari, 2015).

Expression of miRNAs is controlled by the same epigenetic and transcriptional regulatory networks which act on mRNA encoding genes, and therefore is dynamically regulated in response to external stimuli (Avraham and Yarden, 2012). The sequences of many mature miRNAs identified in human (2,588) and mouse (1,915) are evolutionarily conserved (Kozomara and Griffiths-Jones, 2014). Since each miRNA binds both perfect and imperfect mRNA target sequences, any miRNA may potentially bind up to several hundred distinct mRNA targets (Jia et al., 2014). As revealed by system biological approaches, many target mRNAs of a given miRNA encode proteins with related functions (Cora et al., 2017). Therefore, although engagement of a miRNA may result in a rather moderate inhibition of a single target mRNA only, concurrent inhibition of different mRNAs at the same time may serve to regulate cellular properties in a synergistic manner.

By now, in a number of functional studies several miRNAs have been identified as highly important for the differentiation of DCs (Zhou and Wu, 2017). Following stimulation of human (Martinez-Nunez et al., 2009) and mouse (Lu et al., 2011) DCs, a number of miRNAs including miR-155 were shown to be upregulated and were reported to control the immunogenic function of DCs (Smyth et al., 2015). While the decisive role of miRNAs for DC differentiation and activation has been thoroughly demonstrated, the importance of miRNAs for the induction and maintenance of a protolerogenic state in APCs has not been thoroughly analyzed. However, a better knowledge of the miRNAs that contribute to an overall tolerogenic state of APC is highly important for the development of immunotherapies to treat allergies and autoimmune diseases (Loyer et al., 2015) by application of miRNA mimicks or antagomirs (Zhou et al., 2016). In this study, we aimed to identify miRNAs convergingly regulated in differentially tolerized APCs since such miRNAs may constitute key regulators of DC functions.

We show that mouse APCs, differentiated under DC-promoting conditions and tolerized using glucocorticoid or IL-10, commonly down-regulated set some miRNAs as compared with the corresponding control DC population at unstimulated state and after stimulation with LPS (mmu-miR-9-5p, mmu-miR-9-5p, mmu-miR-155-5p). We identified mmu-miR-223-3p as upregulated in both tolerogenic APC populations after stimulation with LPS. We show that overexpression of mmu-miR-223-3p in differentiated DC was sufficient to imprint a maturation-resistant protolerogenic state which was associated with the down-regulation of target mRNAs that contribute to DC-activating cellular pathways.

## Materials and methods

### Animals

Mice [C57BL/6, BALB/c, OT-II (on C57BL/6 background)] were bred and maintained in the Translational Animal Research Center of the University Medical Center Mainz under pathogen-free conditions on a standard diet. The recommendations of the Guide for the Care and Use of Laboratory Animals by the National Institutes of Health (NIH Publications No. 8023, revised 1978) were followed. The Ethics Commission according to the German Animal Welfare Act (Landesuntersuchungsamt of the state Rhineland-Palatinate) approved the use of primary mouse cells for experiments.

### Generation of BMDCs

Bone marrow-derived DCs (BMDCs) were differentiated from bone marrow progenitors of C57BL/6 mice as first described by Scheicher et al. (1992) and modified by Gisch et al. (2007). BM cells (2 × 10^6^ cells/10 ml) were seeded on bacterial dishes (ø 94 mm; Greiner Bio-One, Frickenhausen, Germany) in culture medium [IMDM with 5% FCS (PAA, Cölbe, Germany), 2 mM L-glutamine, 100 U/ml penicillin, 100 μg/ml streptomycin (all from Sigma-Aldrich, Deisenhofen, Germany), and 50 μM ß-mercaptoethanol (Roth, Karlsruhe, Germany)], supplemented with 5% of GM-CSF containing cell culture supernatant derived from X63.Ag8-653 myeloma cells stably transfected with a murine GM-CSF expression construct (Zal et al., 1994) (a kind gift by Dr. B. Stockinger, MRC National Institute for Medical Research, Mill Hill, London) (BMDC culture medium). BMDC culture medium was replenished on days 3 and 6 of culture. In parallel, recombinant murine IL-10 (20 ng/ml; ImmunoTools, Friesoythe, Germany) and dexamethasone (DEX; 1 μM; Sigma-Aldrich, Deisenhofen, Germany) was added on d3 and d6 in the course of replenishment of BMDC culture medium. On d7 of culture, aliquots of derived APC populations were reseeded (10^6^ cells/ml) in wells of 6 well tissue-culture plates (Greiner Bio-One) and stimulated overnight with LPS (1 μg/ml, Merck Millipore, Billerica, MA). On the next day, suspension and loosely adherent cells were harvested, thoroughly washed and subjected to experiments.

### Transient overexpression of mmu-miR-223-3p

For transient overexpression of mmu-miR-223-3p, BMDCs were harvested on d6 of culture and reseeded (10^6^) onto polycarbonate coated transwell inserts (0.8 μm diameter; Corning Costar, Corning, NY) placed into untreated 6 well culture plates (Greiner Bio-One). Then, BMDC culture medium was exchanged against serum-free medium (2 ml). BMDCs were transfected in parallel with each 1 nmol of mmu-miR-223-3p mimick (sense: 5′-UGUCAGUUUGUCAAAUACCCCA-3′; Qiagen, Hilden, Germany) and FITC-labeled scrambled control siRNA (sense: 5′-AGGUCGAACUACGGGUCAAUC-3′; NEB, Ispwich, MA) complexed with GeneSilencer (Genlantis, San Diego, CA). After 4h, medium was exchanged against BMDC culture medium. On the next day, BMDCs were transfected again. On d8, aliquots of transfectants were stimulated with LPS (1 μg/ml) overnight. On the following day, BMDCs were thoroughly washed and used for experiments.

### miRNA array probing and database analysis

Total RNA was isolated from the different APC populations (three experiments: unstimulated APC populations; two experiments: LPS-stimulated APC populations) using the miRNeasy spin-column system (Qiagen, Hilden, Germany) including a DNase treatment step according to the protocol of the manufacturer. RNA quality was checked by measurements on a NanoDrop 2000 spectrophotometer (Thermo Fisher Scientific, Waltham, MA). miRNA probes labeled with Cyanine 3-pCp were generated using the “miRNA Microarray System with miRNA Complete Labeling and Hyb Kit” from Agilent (Santa Clara, CA) using 200 ng of total RNA. Derived fluorescence-labeled RNA was purified and analyzed for quality and yield (Nanodrop ND-100; Peqlab, Erlangen, Germany). Microarrays (Agilent Mouse miRNA Microarray Release 15.0 8x 15K) were hybridized with RNA samples in an Agilent Microarray Hybridization Chamber with fluorescence-labeled RNA using Hi-RPM Hybridization Buffer according to the “miRNA Microarray System with miRNA Complete Labeling and Hyb Kit” from Agilent as recommended by the manufacturer. Afterwards, miRNA array slides were scanned with the Agilent Technologies Scanner G2505B system using Agilent Scan Control software 7.0. Expression data were normalized using Agilent software (background offset 50). miRNA expression in different groups was compared using Chipster v1.4.4 (http://chipster.csc.fi; Kallio et al., 2011) applying two groups test (Bayes; *p*-value threshold 0.05). The results of miRNA array probing were deposited at ArrayExpress (http://www.ebi.ac.uk/arrayexpress; accession number: E-MTAB-6788). The miRNet online ressources (http://www.mirnet.ca; Fan et al., 2016) were used for statistical analysis to identify KEGG pathways significantly affected by mRNAs that were targets of miRNAs convergingly regulated by DEX-APC and IL10-APC, and to visualize miRNA-target mRNA interaction networks.

### Lentivirally mediated overexpression of mmu-miR-223

Cells of the DC-like cell line XS52 (Xu et al., 1995) were cultured in BMDC culture medium, supplemented in addition with 10% of M-CSF containing supernatant of NS47 cells as described. XS52 cells were lentivirally transduced with a copGFP reporter (pGreenFire) or the mmu-miR-223 gene locus (both from SBI Biosciences, Palo Alto, CA) and cultured in the presence of puromycin to select for transductants as described (Bollmann et al., 2014).

### mRNA microarray probing and database analysis

Each 20 μg of total RNA derived from the two transduced XS52 sublines (see above) was reverse-transcribed using the SuperScript Plus Direct cDNA Labeling System (Invitrogen, Carlsbad, CA), with Alexa Fluor 555-aha-dUTP or Alexa Fluor 647-aha-dUTP to differentially label cDNA of either group (two experiments). Derived fluorescence-labeled cDNA was purified and analyzed for quality and yield (Nanodrop ND-100; Peqlab, Erlangen, Germany). Prehybridized (5X SSPE, 0.1% SDS, and 1% BSA) microarrays (Mouse Whole Genome OneArray® Microarray v2; Phalanx Biotech, San Diego) were hybridized with each 20 pmol of either fluorescence-labeled cDNA probe using OneArray Hybridization Buffer with 18% formamide for 16 h (Lucidea SlidePro Hybridizer; GE Healthcare, Munich, Germany) as recommended by the manufacturer. Washed microarrays were analyzed using a ScanArray 4000 system equipped with ScanArray 4.0 software (both Perkin-Elmer, Rodgau-Jügesheim, Germany) at 10 pixel size. Laser intensity (90–100%) and photomultiplier tube sensitivity (60–70%) were varied to optimize signal to background ratios. Derived images were analyzed using the ScanArray 4.0 easyquant software module. This study was performed in accordance with standards defined by the Microarray Gene Expression Data Society (MGED). Expression data have been deposited (http://www.ebi.ac.uk/arrayexpress; accession number: E-MTAB-6369). Intensity data were normalized (loess normalization; MIDAS-2.19) and subjected to significance analysis (MEV 4.3) using appropriate modules of the TM4 microarray software suite (Saeed et al., 2006). Changes in gene expression with *q*-values < 5% and ratios (mmu-miR-223/control) were used for subsequent analyses. The DIANA database Micro-TDS served to identify validated and predicted (threshold set to 0.7) targets of mmu-miR-223-3p (http://diana.imis.athena-innovation.gr/DianaTools/index.php?r=microT_CDS/index; Paraskevopoulou et al., 2016). The PANTHER Pathway 3.4.1 database (http://www.pantherdb.org; Mi et al., 2017) was used to assess the role of validated and putative mmu-miR-223-3p mRNA targets for cellular pathways. Potential interaction of proteins identified as differentially regulated in XS52-mmu-miR-223-3p cells were analyzed using STRING database v10 (http://string-db.org; Szklarczyk et al., 2017).

### Real-time RT-PCR analysis of mature miRNAs and mRNAs

For subsequent detection of mRNA, total RNA was isolated from at least 5 × 10^5^ APCs using the RNeasy MiniPlus kit as recommened (Qiagen, Hilden, Germany), and reverse-transcribed by employing the iScript kit (Bio-Rad, Munich, Germany) as recommended by the manufacturer. Primer pairs used to detect expression of HO-1 (sense: 5′-GAGCCTGAATCGAGCAGAAC-3′, anti-sense: 5′-AGCCTTCTCTGGACACCTGA-3′), were obtained from eurofins MWG Synthesis (Ebersberg, Germany). Sequences of primer pairs employed to detect expression of Gilz and of the house-keeping gene ubiquitin C used for normalization, and performance of real time PCR have been described (Bros et al., 2007). For detection of mature miRNAs, total RNA was isolated using the miRNeasy Mini Kit, reverse-transcribed with the miScript II RT Kit, and subjected to real time PCR using the miScript SYBR Green PCR Kit with miRNA-specific primers as recommended. Expression levels of the small nuclear RNA RNU6B were used for normalization. All kits and primers were obtained from Qiagen.

### Flow cytometry

APCs (5 × 10^5^) were washed in staining buffer (PBS/2% FCS). To block Fc receptor-mediated staining, cells were incubated with rat anti-mouse CD16/CD32 Ab (clone 2.4G2) purified from hybridoma supernatant, for 15 min on ice. Afterwards, cells were incubated with antibodies specific for CD11c (N418, PE-Cy-7), MHCII (M5/114.15.2, PE-Cy5), and CD86 (GL-1, PE). Appropriate isotype controls were employed. All antibodies were of rat origin, and were purchased from Biolegend or eBioscience/Thermo Fisher Scientific (both San Diego, CA). Samples were analyzed using a BD FACS Calibur flow cytometer equipped with CellQuest software (BD Biosciences). Data were analyzed using FlowJo software (FLOWJO, Ashland, OR).

### T cell stimulation assays

C57BL/6 BMDCs (2 × 10^5^/ml) were cocultured with allogeneic splenic BALB/c T cells (3 × 10^6^/ml) for 4 days in a volume of 0.2 ml culture medium for determination of T cell proliferation. T cell proliferation was assessed as genomic incorporation of ^3^H-thymidine (0.25 μCi/well) added for the last 16 h of culture. Cells were harvested onto glass fiber filters and retained radioactivity was measured in a ß counter (1205 Betaplate, LKB Wallac, Turcu, Finnland).

### Statistical analysis

Comparisons of groups were performed by one-way ANOVA using SigmaStat software (SPSS Inc., Chicago, IL). A value of *p* < 0.05 was considered as statistically significant.

## Results

### Comparative analysis of antigen presenting cells tolerized in the course of DC differentiation by applying glucocorticoid and IL-10 reveals a common miRNA expression pattern

Due to the growing evidence that post-transcriptional gene regulation as mediated by miRNAs is essential for proper differentiation and stimulation of DCs, we asked for the relevance of miRNAs for the induction of a maturation-resistant protolerogenic state in DCs. For this, murine BMDC progenitors were cultured under DC-promoting conditions in the presence of GM-CSF to yield immature DCs (iDCs). In parallel assays, aliquots of these were treated with the well established protolerogenic agents DEX (Bros et al., 2007, 2010) and IL-10 (Steinbrink et al., 1999), respectively, starting on day 3 of culture. Aliquots of the derived APC populations were stimulated with LPS.

**Figure 1:**
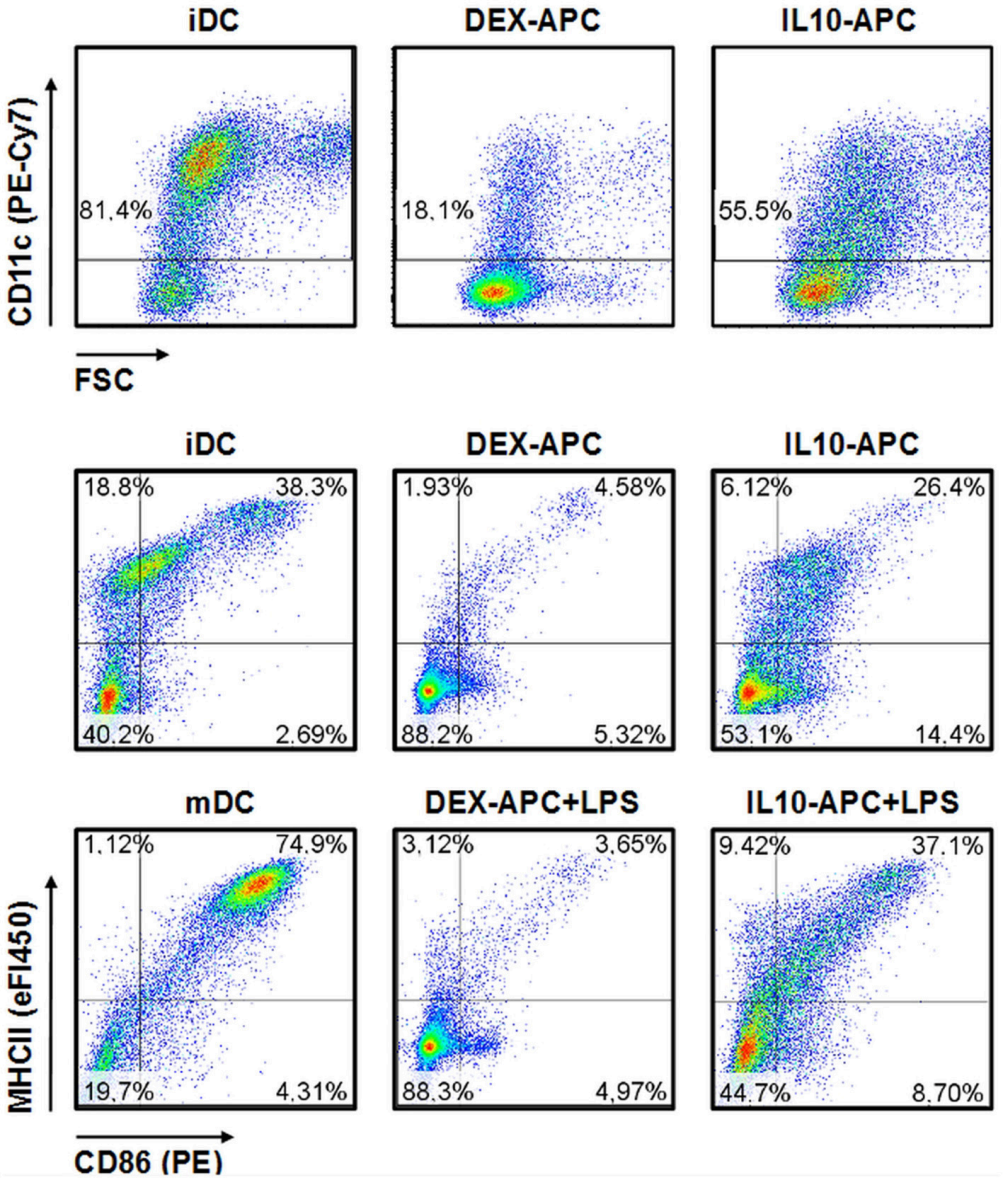
Immuno-phenotype of differentially tolerized APC populations. APC populations were generated as described in the Materials and Methods section, and surface expression of the DC lineage marker CD11c and of the activation markers MHCII and CD86 at unstimulated state and after stimulation with LPS were assessed by flow cytometry. Graphs shown are representative for 2 independent experiments each.

While the vast majority of iDCs expressed the mouse DC lineage marker CD11c, only minor fraction of DEX-APCs and about half of the IL10-APC population expressed this marker (Figure 1). iDC expressed the activation markers MHCII and CD86 at moderate extent. Stimulation with LPS resulted in uniformous upregulation of either marker. On the contrary, only few DEX-APC expressed both markers at either state of activation. IL-10-APC were characterized by moderate expression of MHCII and CD86, below the expression levels observed for iDCs. Treatment with LPS resulted in some upregulation of MHCII while CD86 levels remained largely unaltered.

To confirm that DEX and IL-10 induced differential transcriptional responses in accordingly treated APC populations, we assessed mRNA expression of suitable tolerance markers. The transcription factor Gilz (glucocorticoid-induced leucine zipper) constitutes a genuine target of the activated glucocorticoid receptor and was shown to induce a tolerogenic phenotype in DCs (Calmette et al., 2014). HO-1 was demonstrated to limit the T cell stimulatory capacity of APCs via the degradation product carbon monoxyde which impairs both surface expression of the LPS receptor by interfering with the Golgi apparatus and presentation of antigen via MHCII by blocking endosome maturation (Riquelme et al., 2016). We have previously shown that Gilz is upregulated in differentiating DCs in response to treatment with dexamethasone, whereas HO-1 was induced in response to application of IL-10 (Trojandt et al., 2016). As compared with control DCs, both differentially tolerized APC populations overexpressed mRNAs that encode the aforementioned tolerance-promoting effector molecules in an agent-dependent manner as reflected by elevated Gilz mRNA expression in case of DEX-APCs and enhanced HO-1 mRNA levels in case of IL10-APCs especially after LPS stimulation (Figure S1A).

In agreement with moderate expression of DC activation markers, iDCs stimulated proliferation of allogenic T cells at low extent only, whereas mDCs exerted strong T cell stimulatory capacity (Figure S1B). In accordance with their protolerogenic immuno-phenotype, DEX-APCs and IL10-APCs were poor T cell stimulators at either state of activation as compared with the corresponding control DC population.

**Figure 2:**
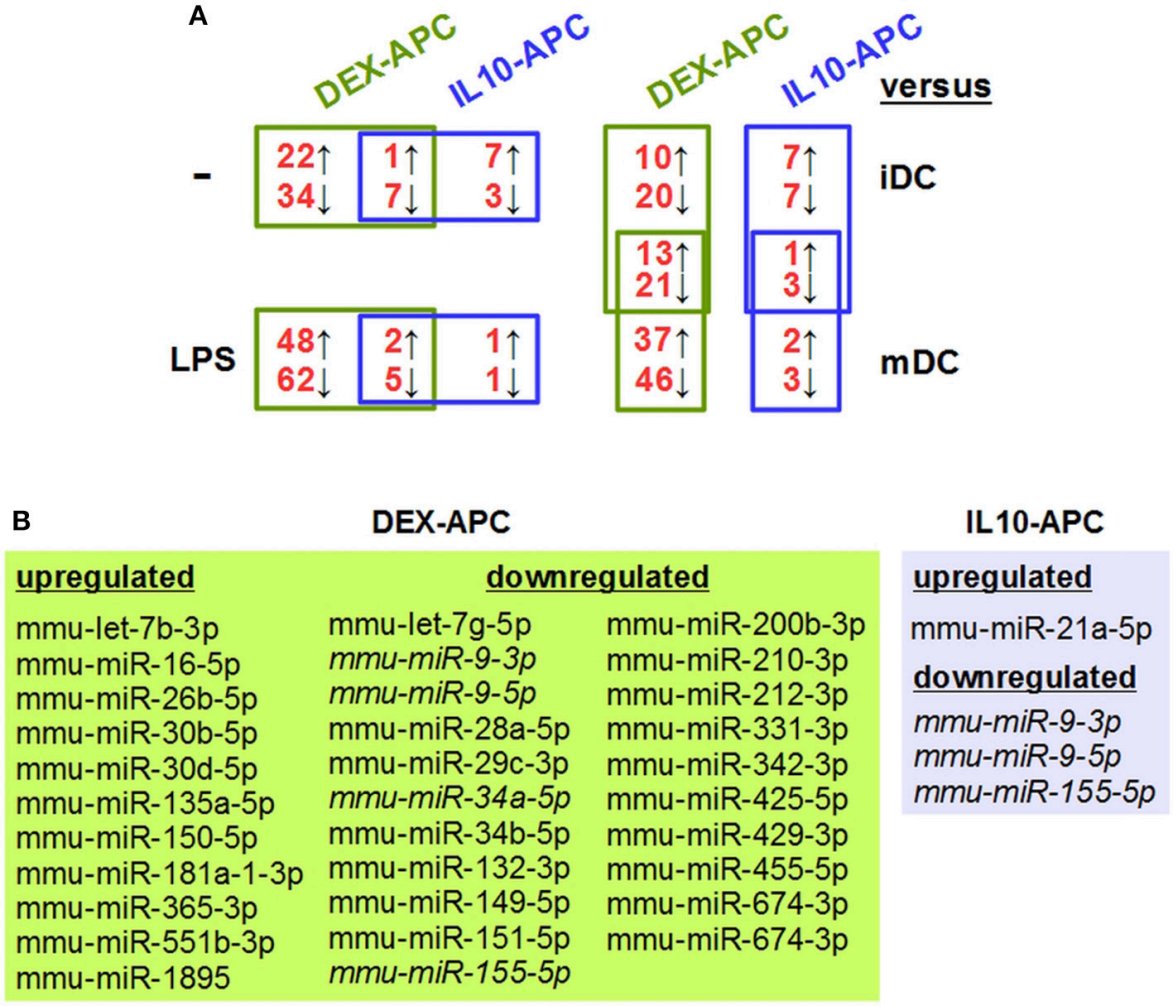
Differentially tolerized APCs at either state of activation largely differ from BMDCs in their miRNA expression signature, but show some overlap. In parallel settings, BMDCs and tolerogenic APC populations (DEX-APC, IL10-APC) were differentiated from the same bone marrow progenitors in the presence of GM-CSF, and the miRNA expression profiles of all APC populations at unstimulated state and after stimulation with LPS were assessed by array analysis in 2 independent experiments. **(A)** The numbers of miRNAs differentially expressed in tolerogenic APCs (DEX-APC, IL10-APC) as compared with BMDCs (iDC, mDC) are indicated (see Tables 1, 3, Tables S1, S2, S7, S8). miRNAs convergingly regulated in differentially tolerized APCs at unstimulated (see Table 1) and at stimulated state (see Table 3) as compared with the according control DC population (iDCs, mDCs) are listed in the assigned tables. **(B)** miRNAs convergingly regulated in differentially tolerized APCs as compared with the corresponding control DC population (iDCs, mDCs) at both states of stimulation. miRNAs commonly regulated by both APC populations at both states of stimulation are given in italics.

In order to identify tolerance-associated miRNAs, we performed miRNA array analysis on DEX-APCs and IL10-APCs at unstimulated state and after stimulation with LPS and included the corresponding control DC populations (iDC, mDC). Unstimulated DEX-APCs (23 upregulated, 41 downregulated; Table S1) and IL10-APCs (8 upregulated, 10 downregulated; Table S2) differentially expressed a number of miRNAs as compared with iDCs (Figure 2A, left upper panel). Interestingly, the differential miRNA expression pattern of DEX-APCs (Table S3) and IL10-APCs (Table S4) was predicted to affect a largely overlapping set of biological processes, including MAPK, PI3K, and Wnt signaling pathways, which are known to affect the DC activation state, as well as the actin cytoskeleton required by DCs for antigen uptake, migration and interaction with T cells.

Both differentially tolerized APC populations convergingly overexpressed mmu-miR-27b-3p, and were characterized by diminished expression of 7 miRNAs as compared with iDCs (Table 1). Cellular pathways predicted to be altered by differential expression of the convergingly regulated miRNAs are listed in Table 2. Many of the signaling pathways are known to promote (e.g., MAPK, NF-κB, Ras) or inhibit (e.g., cAMP, Wnt) DC activation. miRNA:mRNA target network analysis showed that in case of unstimulated tolerized APCs the convergingly upregulated mmu-miR-27b-3p interacted with commonly down-regulated miRNA species in 11 of 26 signaling pathways potentially regulated (Figure S2).

**Table 1:** miRNA species convergingly regulated in DEX-APCs and IL10-APCs at unstimulated state in comparison with iDCs as detected by miRNA array analysis.

**miRNA**	**DEX-APC**		**IL-10-APC**	
	**Fold Change (log_2_ vs. iDC)**	***p*-value**	**Fold change (log_2_ vs. iDC)**	***p*-value**
mmu-miR-27b-3p	2.03	4.37E-2	1.16	9.43E-3
mmu-miR-130b-3p	−1.39	2.75E-3	−1.12	1.68E-2
mmu-miR-92a-3p	−1.40	8.16E-3	−1.16	1.33E-3
mmu-miR-146a-5p	−2.40	8.07E-4	−1.22	8.38E-3
mmu-miR-151-5p	−2.15	5.61E-4	−1.37	4.00E-6
mmu-miR-9-3p	−2.72	4.00E-6	−1.53	1.00E-6
mmu-miR-9-5p	−3.77	5.00E-6	−1.71	1.00E-6
mmu-miR-155-5p	−11.63	8.00E-6	−1.60	7.00E-6

**Table 2:** KEGG pathways potentially affected by miRNA species convergingly regulated in DEX-APCs and IL10-APCs at unstimulated state as compared with iDCs.

**KEGG pathway**	**# genes^a^**	***p*-value**
**SIGNAL TRANSDUCTION**		
cAMP signaling pathway (mmu4027)	25	1.96E-6
Toll-like receptor signaling pathway (mmu04620)	17	3.33E-6
mTOR signaling pathway (mmu04150)	13	5.43E-6
FoxO signaling pathway (mmu04068)	19	7.86E-6
MAPK signaling pathway (mmu04010)	26	2.84E-5
Chemokine signaling pathway (mmu04062)	22	4.21E-5
AMPK signaling pathway (mmu04152)	17	5.67E-5
cGMP-PKG signaling pathway (mmu04022)	19	1.61E-4
Fc epsilon RI signaling pathway (mmu04664)	11	2.29E-4
Sphingolipid signaling pathway (mmu04071)	15	3.43E-4
TNF signaling pathway (mmu04668)	14	3.43E-4
Rap1 signaling pathway (mmu04015)	20	6.90E-4
Ras signaling pathway (mmu04014)	20	1.61E-3
NF-kappa B signaling pathway (mmu04064)	12	1.61E-3
VEGF signaling pathway (mmu04370)	9	1.61E-3
Phosphatidylinositol signaling system (mmu04070)	11	3.51E-3
Wnt signaling pathway (mmu04310)	14	3.51E-3
Jak-STAT signaling pathway (mmu04630)	15	3.54E-3
PI3K-Akt signaling pathway (mmu04151)	25	4.44E-3
HIF-1 signaling pathway (mmu04919)	11	7.71E-3
Phospholipase D signaling pathway (mmu04072)	13	8.62E-3
TGF-beta signaling pathway (mmu04350)	9	1.31E-2
Hippo signaling pathway (mmu04390)	13	1.46E-2
Hedgehog signaling pathway (mmu04341)	6	2.38E-2
Notch signaling pathway (mmu04330)	6	2.60E-2
RIG-I-like receptor signaling pathway (mmu04622)	7	3.54E-2
**CELL STRUCTURE/MOTILITY**		
Focal adhesion (mmu04510)	20	4.94E-4
Adherens junction (mmu04520)	10	1.70E-3
Leukocyte transendothelial migration (mmu04670)	13	2.29E-3
Tight junction (mmu04530)	13	7.03E-3
Amoebiasis (mmu05146)	11	1.35E-2
Gap junction (mmu04540)	8	3.93E-2
**UPTAKE**		
Fc gamma R-mediated phagocytosis (mmu04666)	12	5.08E-4
Endocytosis (mmu04144)	23	1.24E-3
**METABOLISM**		
Apoptosis (mmu04215)	14	1.96E-5
Inositol phosphate metabolism (mmu00562)	10	1.20E-3
Biosynthesis of unsaturated fatty acids (mmu01040)	6	1.72E-3
Carbohydrate digestion and absorption (mmu04973)	7	4.44E-3
Fatty acid metabolism (mmu01212)	6	3.12E-2

a *Number of mRNA species within the given pathway potentially affected by the miRNA species convergingly regulated in tolerized APC populations*.

In response to stimulation with LPS, mDCs upregulated 45 miRNA species as compared with iDCs, including mmu-miR-155-5p and mmu-miR-146a-5p which are well known stimulation-associated miRNAs in DCs, and down-regulated 31 miRNA species (Table S5). These alterations are predicted to affect several signaling pathways (e.g., PI3K-AKT, MAPK, Wnt, chemokine) and cellular functions (e.g., endocytosis, actin cytoskeleton, protein processing) (Table S6), which are strongly altered in the course of DC activation. DEX-APCs (Table S7) and IL10-APCs (Table S8) responded to LPS stimulation with differential expression of a number of miRNAs. Similar to the miRNA expression patterns at unstimulated state, after LPS stimulation DEX-APCs were characterized by a larger number of differentially regulated miRNA (50 up, 67 down) as compared with mDCs than IL10-APCs (3 up, 6 down) (Figure 2A, left lower panel). Subsets of miRNAs were differentially regulated by either APC population as compared with the corresponding control DC population (iDCs, mDCs) at both states of activation (Figures 2A, right panel, B). The miRNA signature of stimulated DEX-APCs as compared with mDCs (Table S9) was predicted to affect in most cases the same biological processes as observed for DEX-APCs compared with iDCs (see Table S3). In case of IL10-APCs, an overall similar pattern of potentially affected pathways as compared with the corresponding control DC population was observed, although the number of signal transduction pathways was lower in case of LPS-stimulated IL10-APCs (Table S10, see Table S4).

**Figure 3:**
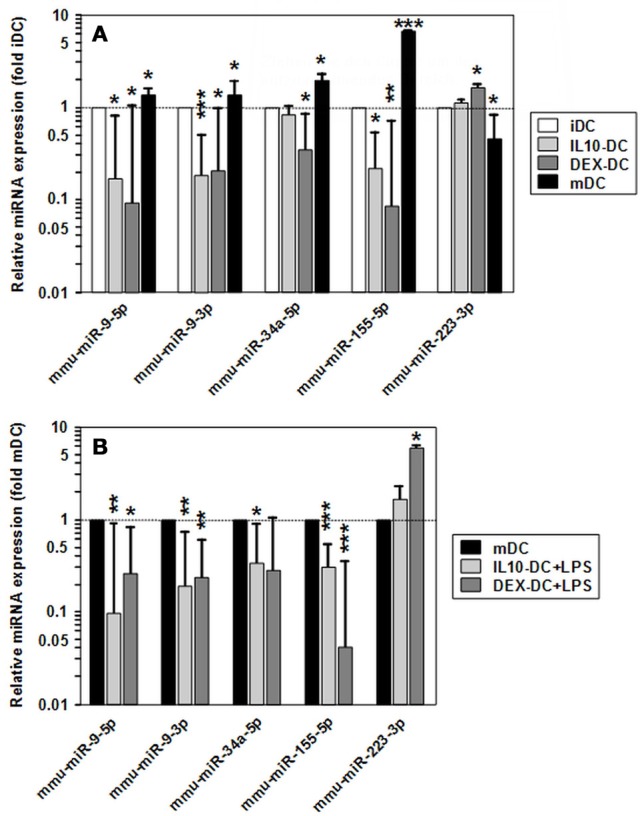
Validation of differential miRNA expression in tolerized APCs vs. BMDCs. Levels of mature miRNA expression in tolerized APC populations and BMDCs at **(A)** unstimulated and **(B)** LPS-stimulated state were assessed by real time PCR. To indicate stimulation-dependent alterations in miRNA expression in BMDCs, according levels in mDCs as compared with iDCs are given in **(A)**. **(A,B)** Data indicate the mean ± SEM of 4 independent experiments performed in duplicate. Statistically significant differences: *vs. corresponding control DC population (iDC,mDC) (*p < 0.05, **p < 0.01, ***p < 0.001).

Among the panels of LPS-regulated miRNAs, as compared with mDCs both tolerogenic APC populations regulated a number of miRNAs convergingly (Figure 2A, left lower panel; Table 3). Of these, mmu-miR-223-3p and mmu-miR-1224-5p were upregulated in the stimulated tolerogenic APC populations, and a panel of 5 miRNAs was down-regulated. Interestingly, this set of miRNA species was regulated conversely in mDCs vs. iDCs. Within the panel of miRNAs down-regulated in the tolerogenic APC populations after LPS stimulation, mmu-miR-9-3p, mmu-miR-9-5p, and mmu-miR-155-5p were also diminished in expression in tolerogenic APCs at unstimulated state (see Figure 2B and Table 1). The target mRNAs of miRNAs convergingly regulated in the LPS-stimulated tolerogenic APC populations were predicted to affect a panel of cellular pathways which largely overlap with those predicted as altered in both tolerogenic APC populations at unstimulated state (Table 4; compare Table 2). miRNA-mRNA target interaction network analysis showed that mmu-miR-223-3p potentially interacted with commonly down-regulated miRNAs in all potentially affected signaling pathways (not shown). In contrast, validated targets of mmu-miR-1224-5p were not coregulated by any other convergingly regulated miRNA. Real time-PCR analysis confirmed differential expression of selected miRNAs in the different APC populations at unstimulated state (Figure 3A) and after stimulation with LPS (Figure 3B).

**Table 3:** miRNA species convergingly regulated in DEX-APCs and IL10-APCs at stimulated state in comparison with mDCs as detected by miRNA array analysis^a^.

**miRNA**	**DEX-APC** + **LPS**		**IL-10-APC** + **LPS**		**mDC**	
	**Fold change (log_2_ vs. mDC)**	***p*-value**	**Fold change (log_2_ vs. mDC)**	***p*-value**	**Fold Change (log_2_ vs. iDC)**	***p*-value**
mmu-miR-223-3p	2.77	1.00E-06	3.43	4.45E-02	−1.27	2.06E-04
mmu-miR-1224-5p	2.44	1.00E-06	4.45	4.45E-02	−1.63	1.00E-06
mmu-miR-29a-3p	−1.52	1.00E-06	−2.114	4.19E-02	1.31	1.00E-06
mmu-miR-34a-5p	−1.57	1.00E-06	−1.982	4.19E-02	1.28	1.00E-06
*mmu-miR-9-3p*	−1.72	1.00E-06	−3.454	4.45E-02	1.10	1.30E-04
*mmu-miR-9-5p*	−2.00	1.00E-06	−3.564	1.52E-02	1.12	1.26E-03
*mmu-miR-155-5p*	−3.35	1.00E-06	−4.891	4.19E-02	1.80	1.00E-06

a *miRNAs convergingly regulated in tolerized APC populations at unstimulated state (see Table 1) and after LPS treatment are given in italics*.

**Table 4:** KEGG pathways potentially affected by miRNA species convergingly regulated in DEX-APCs and IL10-APCs at stimulated state as compared with mDCs.

**KEGG pathway**	**#genes^a^**	***p*-value**
**SIGNAL TRANSDUCTION**		
PI3K-Akt signaling pathway (mmu04151)	49	4.11E-9
mTOR signaling pathway (mmu04150)	16	1.05E-6
cAMP signaling pathway (mmu4027)	29	4.91E-6
FoxO signaling pathway (mmu04068)	23	4.91E-6
Rap1 signaling pathway (mmu04015)	29	1.35E-5
AMPK signaling pathway (mmu04152)	21	2.02E-5
Ras signaling pathway (mmu04014)	28	1.11E-4
Fc epsilon RI signaling pathway (mmu04664)	13	1.84E-4
MAPK signaling pathway (mmu04010)	29	1.97E-4
TNF signaling pathway (mmu04668)	17	2.26E-4
Sphingolipid signaling pathway (mmu04071)	18	2.80E-4
cGMP-PKG signaling pathway (mmu04022)	22	2.94E-4
Chemokine signaling pathway (mmu04062)	23	7.10E-4
Toll-like receptor signaling pathway (mmu04620)	15	7.34E-4
VEGF signaling pathway (mmu04370)	11	8.20E-4
Phospholipase D signaling pathway (mmu04072)	18	1.46E-3
HIF-1 signaling pathway (mmu04919)	14	4.48E-3
Jak-STAT signaling pathway (mmu04630)	18	4.82E-3
Hippo signaling pathway (mmu04390)	16	1.59E-2
Phosphatidylinositol signaling system (mmu04070)	11	2.92E-2
Notch signaling pathway (mmu04330)	7	3.26E-2
Wnt signaling pathway (mmu04310)	14	4.00E-2
**CELL STRUCTURE/MOTILITY**		
Focal adhesion (mmu04510)	41	5.70E-12
Leukocyte transendothelial migration (mmu04670)	18	2.26E-4
Amoebiasis (mmu05146)	17	4.53E-4
Regulation of actin cytoskeleton (mmu04810)	25	4.90E-4
Tight junction (mmu04530)	17	2.77E-3
Adherens junction (mmu04520)	11	4.48E-3
ECM-receptor interaction (mmu04512)	12	5.56E-3
Gap junction (mmu04540)	10	3.27E-2
**UPTAKE**		
Endocytosis (mmu04144)	31	1.84E-4
Fc gamma R-mediated phagocytosis (mmu04666)	14	6.04E-4
**METABOLISM**		
Apoptosis (mmu04215)	14	2.8E-4
Protein digestion and absorption (mmu04974)	14	7.34E-4
Carbohydrate digestion and absorption (mmu04973)	8	6.05E-3
Inositol phosphate metabolism (mmu00562)	9	2.58E-2
Biosynthesis of unsaturated fatty acids (mmu01040)	5	3.15E-2

a *Number of mRNA species within the given pathway potentially affected by the miRNA species convergingly regulated in tolerized APC populations*.

Altogether, our findings strongly suggest that the aforementioned panel of seven miRNAs which on one hand are convergingly regulated in both protolerogenic APC populations after LPS treatment as compared with mDCs (see Table 3) may contribute to establish and maintain a protolerogenic state in APCs. The observation of converse regulation of this set of miRNAs in mDCs vs. iDCs suggests that their down-regulation may be essential for DC maturation. mmu-miR-9-3p, mmu-miR-9-5p, and mmu-miR-155-5p were attenuated in expression in both tolerogenic APC populations at either state of activation as compared with the corresponding control DC population which may underscore their importance for the imprintment of a protolerogenic state. Of the two miRNA species convergingly upregulated in tolerized APCs at stimulated state as compared with mDCs, mmu-miR-223-3p is of considerable interest due to the predicted contribution of its mRNA targets to numerous signaling pathways that determine the state of DC activation.

### Overexpression of mmu-miR-223-3p in DCs prevents the acquisition of stimulation-associated potent T cell stimulatory activity

**Figure 4:**
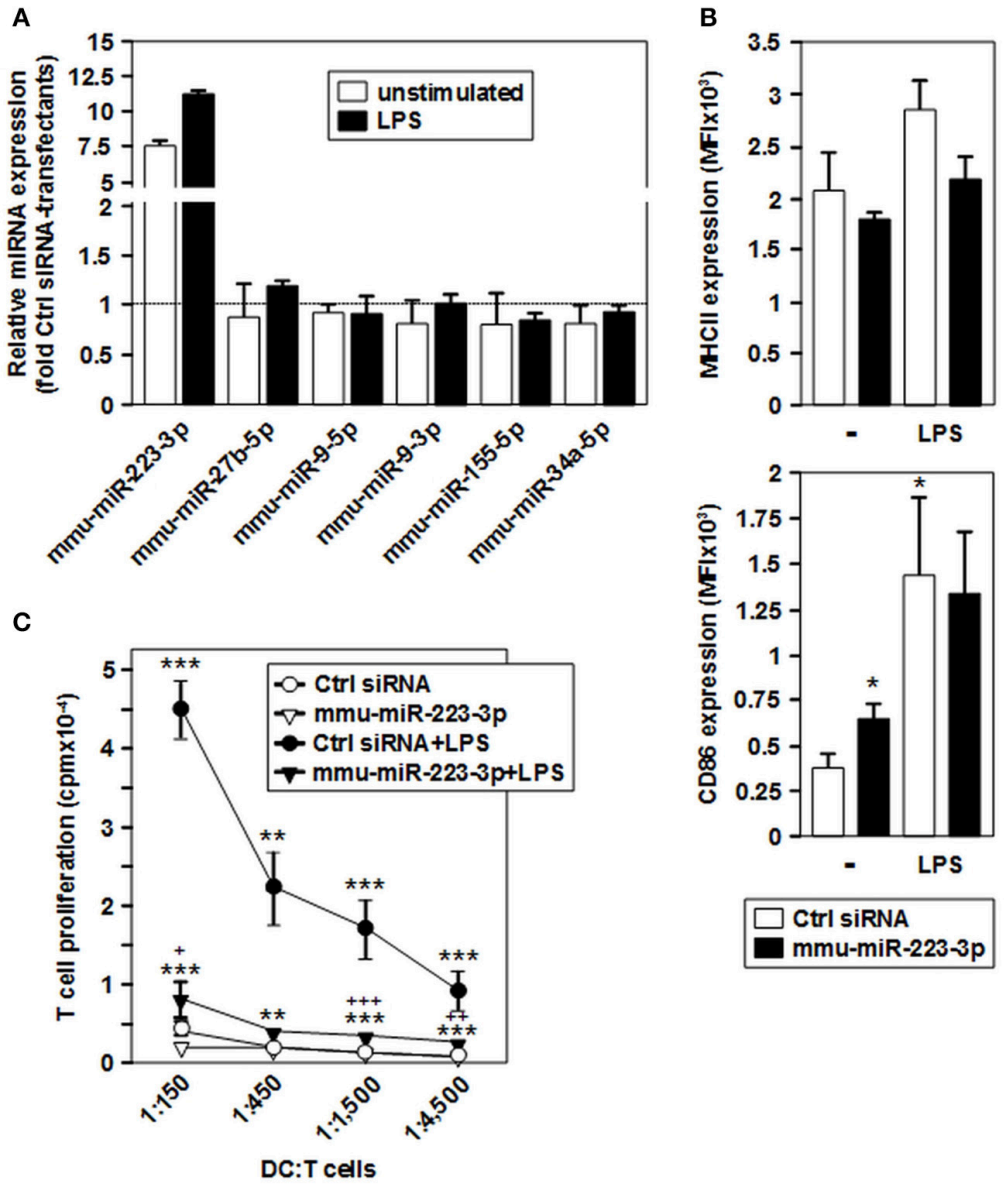
Overexpression of mmu-miR-223-3p in BMDCs prevents stimulation-induced upregulation of MHCII and the acquisition of potent T cell stimulatory capacity. iDCs were transfected with a mmu-miR-223-3p mimick or a scrambled siRNA as a control, and aliquots were stimulated with LPS. **(A)** Relative expression levels of mmu-miR-223-3p and other miRNA species in mmu-miR-223-3p-transfected BMDC populations as compared with Ctrl sirRNA-transfectants were assessed by real time PCR analysis. Data indicate the mean ± SD of 2 independent experiments performed in duplicate. **(B)** Surface expression of MHCII (upper panel) and CD86 (lower panel) in differentially transfected BMDCs at either state of activation was monitored by flow cytometry. Data indicate the mean fluorescence intensity (MFI) ±SEM of 5 independent experiments each. **(C)** Differentially transfected BMDC populations were titrated (starting concentration: 2 × 10^5^/ml) and cocultured with allogeneic T cells (each 3 × 10^6^/ml) in triplicates at the indicated ratios for 4 days. Data indicate T cell proliferation quantitated as cpm due to genomic incorporation of ^3^H-thymidine applied for the last 16–18 h of coculture, and denote the mean ± SEM of 5 independent experiments performed in triplicate. **(B,C)** Statistically significant differences: *vs. Ctrl siRNA, and + vs. mmu-miR-223-3p transfected BMDC at unstimulated state (*^, +^*p* < 0.05, **^, ++^*p* < 0.01, ***^, +++^*p* < 0.001).

To evaluate the effect of mmu-miR-223-3p overexpression on differentiated DCs, we transfected iDCs with a mmu-miR-223-3p mimic. Aliquots of transfectants were stimulated with LPS. To determine the transfection efficiency, a FITC-labeled control siRNA was used in parallel experiments, and the frequency of FITC-positive cells was assessed by flow cytometry (60–78%; Figure S3) prior to experimental use. Transfection of iDCs with mmu-miR-223-3p resulted in strong overexpression of this miRNA species (~12-fold) as compared with control siRNA-transfected iDCs, and remained on similar level in response to stimulation with LPS (Figure 4A). Expression of other miRNA species identified as differentially regulated in the tolerized APC populations remained unaltered which indicates that mmu-miR-223-3p exerts no direct regulatory effect on their expression. iDCs transfected with mmu-miR-223-3p showed comparable expression of the antigen presentation receptor MHCII (Figures 4, 3B, upper panel) and somewhat elevated levels of the costimulatory marker CD86 (Figures 4, 3B, lower panel) as compared with control siRNA-transfectants. Stimulation with LPS resulted in moderate upregulation of MHCII by control DC, while mmu-miR-223-3p transfectants failed to substantially upregulate MHCII. On the other hand, both DC populations comparably enhanced CD86 expression in response to LPS stimulation. Furthermore, supernatants of LPS-stimulated DC populations contained comparable levels of cytokines (Figure S4).

In agreement with low expression of MHCII and CD86, iDCs transfected with mmu-miR-223-3p and control siRNA exerted only low allogeneic T cell stimulatory activity (Figure 4C). LPS-stimulated control-transfected DCs which displayed upregulated expression of both markers mediated profound T cell proliferation. On the contrary, when stimulated with LPS, mmu-miR-223-3p overexpressing DCs induced only weak T cell proliferation at similar level as did the unstimulated DC populations. The impaired T cell proliferation in cocultures using LPS-stimulated DCs transfected with mmu-miR-223-3p was not associated with an increase in apoptotic T cells or Foxp3^+^ T cells (not shown). LPS-stimulated miR-223 transfectants loaded with OVA peptide were also poor stimulators of OVA peptide-specific CD4^+^ OTII T cells (Figure S5).

These observations suggest that overexpression of mmu-miR-223-3p is sufficient to establish a maturation-resistant tolerogenic state in DC.

### Stable overexpression of miR-223-3p in a DC line attenuates expression of several validated target mRNA species whose products regulate DC activating signaling pathways

Due to our finding of a mmu-miR-223-3p-induced functional arrest of DCs, we next asked for its mRNA targets. To obtain a homogenous population of miR-223-3p overexpressing DCs, we stably overexpressed mmu-miR-223-3p in the DC-like cell line XS52. XS52 cells lentivirally transduced with a GFP reporter served as a control. One week after selection, all transductants were GFP-positive (not shown), and accordingly transduced XS52 cells expressed mmu-miR-223-3p at ca. 4-fold higher level than the GFP expressing control subline (not shown). Transcriptome analysis revealed differential expression of 155 mRNA species [up (>2-fold): 92, down (< 0.5-fold): 63] in mmu-miR-223-3p overexpressing XS52 as compared with the control subline (Table S11).

**Figure 5:**
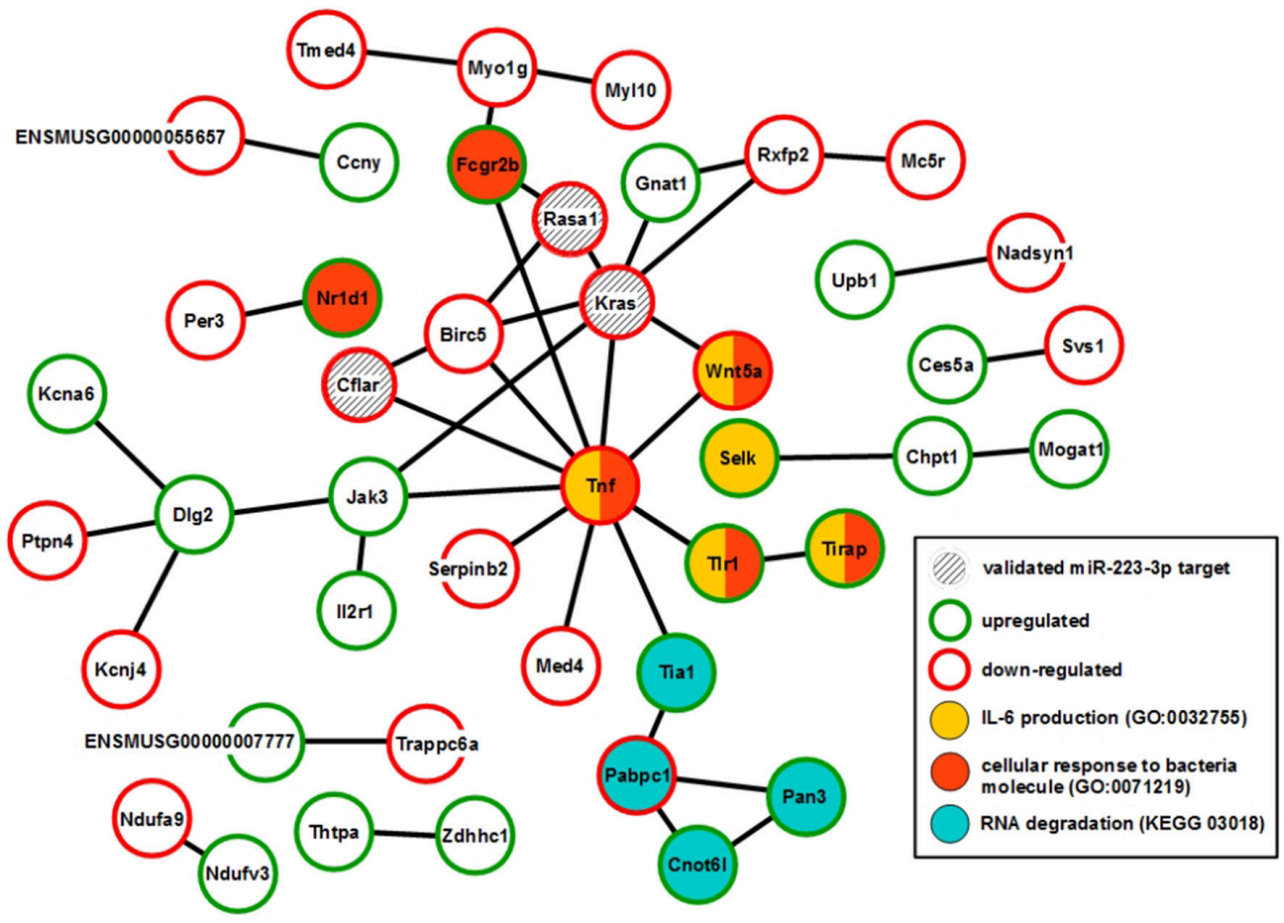
Overexpression of mmu-miR-223-3p in DCs results in reduced mRNA expression of its genuine targets Rasa1 and Kras and of other mRNA species which may affect a network of immunologically relevant proteins. Database analysis of the mRNA expression pattern of DC-like XS52 cells stably transduced to overexpress mmu-miR-223-3p (see Table S11) predicted networks of the encoded proteins. The state of expression of corresponding mRNA species in mmu-miR-223-3p overexpressing XS52 cells as compared with the vector control transduced subline is indicated. The genuine (Kras, Rasa1, Cflar, Fbxl20) mRNA targets of mmu-miR-223-3p are accordingly labeled. Potentially interacting proteins that share immunological and gene regulatory functions are color-coded.

XS52 cells overexpressing mmu-miR-223-3p expressed the validated mmu-miR-223-3p mRNA targets Kras (Kirsten rat sarcoma viral oncogene homolog; Minato, 2013), Rasa1 (RAS p21 protein activator 1; King et al., 2013), Cflar (CASP8 and FADD-like apoptosis regulator; Wittkopf et al., 2013), and Fbxl20 (F-box and leucine-rich repeat protein 20; Xiao et al., 2015) as well as the predicted mmu-miR-223-3p mRNA target Cmtm3 (CKLF-like MARVEL transmembrane domain containing 3; Li et al., 2014) at lower extent than the control subline. Of these mRNAs, only Cflar, Kras, and Rasa1 affect biological processes potentially altered in mmu-miR-223-3p overexpressing DC (Table S12). Furthermore, the differentially expressed mRNAs in miR-223-3p overexpressing DCs are allocated to a total of 25 cellular pathways relevant in immune cells. Within the group of regulated mmu-miR-223-3p targets, Rasa1 and Kras each contributes to 13 of these, and Cflar is involved in two pathways (Table 5). The other mmu-miR-223-3p targets have not been attributed any function within these pathways yet. Protein network analysis based on the transcriptional signature of miR-223-3p overexpressing XS52 revealed potential interaction of Rasa1, Kras, and Cflar (Figure 5). Moreover, several other proteins within that predicted network are overlappingly involved in bacteria-induced cellular responses and IL-6 production (Tirap, Tlr1, TNF, Wnt5a), while another group of proteins described to interact contributes to the control of mRNA stability (Cnot6l, Pabpc1, Pan3, Tia1).

**Table 5:** Biological pathways potentially affected by overexpression of mmu-miR-223-3p in DCs.

**Pathway**	**No. of genes^a^**	**Included in pathway control**		
		**Kras**	**Rasa1**	**Cflar**
Angiogenesis (P00005)	4	•	•	
Interleukin signaling pathway (P00036)	3		•	
PDGF signaling pathway (P00047)	3	•	•	
Apoptosis signaling pathway (P00006)	2			•
EGF receptor signaling pathway (P00018)	2	•	•	
Fas signaling pathway (P00020)				•
FGF signaling pathway (P00021)	2	•	•	
Gonadotropin-releasing hormone receptor pathway (P06664)	2			
Ras Pathway (P04393)	2	•	•	
TGF-beta signaling pathway (P00052)	2	•		
Toll receptor signaling pathway (P00054)	2			
Wnt signaling pathway (P00057)	2			
Cadherin signaling pathway (P00012)	1			
FAS signaling pathway (P00020)	1			
General transcription regulation (P00023)	1			
Inflammation mediated by chemokine and cytokine signaling pathway (P00031)	1	•		
Insulin/IGF pathway-mitogen activated protein kinase kinase/MAP kinase cascade (P00032)	1		•	
Integrin signaling pathway (P00034)	1	•		
p53 pathway feedback loops 2 (P04398)	1	•		
Phenylethylamine degradation (P02766)	1			
PI3 kinase pathway (P00048)	1	•		
Pyrimidine Metabolism (P02771)	1			
Pyruvate metabolism (P02772)	1			
Transcription regulation by bZIP transcription factor (P00055)	1			
Ubiquitin proteasome pathway (P00060)	1			
VEGF signaling pathway (P00056)	1	•		

a *Number of mRNA species within the pathway potentially affected upon mmu-miR-223-3p overexpression*.

Altogether, our study has identified sets of miRNAs that are commonly regulated by differentially tolerized APCs. We identified mmu-miR-223-3p as convergingly upregulated in tolerogenic stimulated APCs as compared with mDCs, and its overexpression was sufficient to imprint a maturation-resistant state of activity in BMDCs. Stable overexpression of mmu-miR-223-3p in a DC-like cell line was found to diminish expression of target mRNAs which contribute to signaling pathways relevant for the DC activation state, including Ras signaling.

## Discussion

DCs play a crucial role in the regulation of adaptive immune responses due to their high degree of functional plasticity: immature DC continuously engulf material from their micro-environment, and present derived antigen fragments in a non-immunogenic context to induce anergy, apoptosis or a regulatory state in antigen-specific T cells (Bardhan et al., 2016; Devi and Anandasabapathy, 2017). In contrast, DCs activated by pathogen-derived or endogenous danger signals mature and acquire strong T cell stimulatory capacity (Joffre et al., 2009). To prevent excessive and chronic inflammation that may result in the arisal of autoimmune diseases, regulatory T cells, but also activated DCs generate antiinflammatory mediators like IL-10 that contribute to resolve inflammation (MacKenzie et al., 2014). Other endogenous tolerance-promoting mediators like glucocorticoids dampen the overall activation state of immune cells to prevent autoimmunity (Cain and Cidlowski, 2017). In accordance, glucocorticoids are frequently used for corresponding treatment.

By now, the importance of miRNAs for the differentiation of hematopoietic cells like DCs, and their activation-induced regulation has been well established (Forero et al., 2017). In this regard, mmu-miR-155-5p was the first miRNA identified to play an essential role for the activation of DCs and other APC populations (Rodriguez et al., 2007). However, the role of miRNA species in establishing a tolerogenic state in DCs has not been thoroughly addressed so far.

Here we asked whether different protolerogenic agents may imprint a common miRNA signature in maturation-resistant APCs. For this, we used the glucocorticoid DEX and the prototypic antinflammatory cytokine IL-10 to generate tolerogenic APCs when applied to bone marrow cells differentiated under BMDC-promoting conditions using GM-CSF. Derived BMDCs are composed of conventional DCs differentiated from distinct progenitor cells, and cultures contain some macrophages (Helft et al., 2015; Lutz et al., 2016). The only alternative well established DC differentiation protocol starting with total bone marrow cells employs FLT3 ligand yielding a mixture of lymphoid and conventional DC subpopulations, at much lower cell number than obtained using GM-CSF (Weimershaus and van Endert, 2013). Moreover, FLT3 ligand was demonstrated to promote the differentiation of non-DC populations (Dannaeus et al., 1999).

As previously shown by us and others APC populations differentiated from bone marrow using GM-CSF plus either DEX or IL-10 display impaired expression of costimulators and of MHCII, and are largely resistant to maturation in terms of activation marker expression, and T cell stimulatory capacity (Steinbrink et al., 1999; Bros et al., 2007). We observed that IL10-APCs were characterized by altered expression of a limited number of miRNAs at either state of stimulation, while DEX-APCs showed a more extensively altered miRNA expression profile as compared with the corresponding control DC population. This marked difference in the extent of miRNA regulation may be explained by the differential intracellular signaling evoked by both agents: IL-10 largely acts via activation of the JAK/STAT3 pathway which results in expression of STAT3 target genes (Mosser and Zhang, 2008). In addition, STAT3 interacts with NF-κB p65 (Bode et al., 2012) and transcription factors of the FOXO family (Oh et al., 2012) to affect expression of their target genes. Glucocorticoids activate the cytosolic glucocorticoid receptor (GR), which itself acts as a transcription factor that elicits expression of glucocorticoid-responsive genes (Weikum et al., 2017). Moreover, activated GR was demonstrated to interact with numerous other transcription factors including NF-κB (Bekhbat et al., 2017), and thereby to affect the expression of numerous distinct target genes. Since expression of miRNAs is controlled by the same transcription factors that regulate mRNA expression (Avraham and Yarden, 2012), our findings suggest that the activated GR regulates more miRNAs than activated STAT3.

We show that APCs tolerized by long term treatment with IL-10 or DEX under DC-promoting conditions commonly overexpressed mmu-miR-27b-3p as compared with iDC, while a set of 7 miRNAs was convergingly down-regulated. Interestingly, mmu-miR-27b-3p was previously shown to be upregulated by DEX *in vivo*, and to inhibit browning of white adipose tissue by inhibiting Prdm16 (Kong et al., 2015). In favor of a potential anti-inflammatory role, mmu-miR-27b-3p was demonstrated to target PPAR-γ mRNA, and thereby to attenuate PPAR-γ-mediated NF-κB activation in neuroblastoma cells (Lee et al., 2012). mmu-miR-27b-3p was found strongly upregulated in allo-transplantated adipose-derived mesenchymal stem cells when accepted by the recipient as compared with rejections which underscores its tolerance-promoting importance (Chen et al., 2013).

We identified also several miRNAs including mmu-miR-146a-5p and mmu-miR-155-5p which were downregulated in differentially tolerized APCs as compared with iDCs, and upregulated in the course of DC stimulation (mDCs vs. iDCs) which suggested that these miRNAs may contribute to the responsiveness of DCs toward stimulation. However, concerning mmu-miR-146a-5p, an opposite role has been demonstrated. This miRNA was reported to be upregulated in immune cells by stimulatory agents like LPS and endogenous proinflammatory mediators via NF-κB and JNK signaling (Perry et al., 2009), including DCs (Karrich et al., 2013). Upregulated miR-146a-5p was shown to act as a negative feed-back regulator of both pathways, in an effort to dampen the magnitude of the immune response by targeting signal adaptors, e.g., of downstream NF-κb signaling including IRAK1 and TRAF6 (Park et al., 2015). Consequently, prolonged expression of miR-146a-5p has been linked to immune tolerance (reviewed in Saba et al., 2014). Similar to miR-146a, miR-155-5p is induced by inflammatory signaling (reviewed in Turner et al., 2011). Similar to our finding of down-regulated mmu-miR-155-5p expression in DEX-APCs and IL10-APCs, mouse macrophages treated with IL-10 in the course of LPS stimulation expressed mmu-miR-155-5p at attenuated extent, in a STAT3-dependent manner (McCoy et al., 2010). Likewise, glucocorticoids were reported to interfere with LPS-induced mmu-miR-155-5p upregulation in macrophages via inhibition of NF-κB signaling (Zheng et al., 2012).

Similar to miR-155-5p, mmu-miR-9-5p was demonstrated to activate NF-κB as shown for microglial cells, and to promote the production of proinflammatory cytokines by targeting MCPIP1 (Yao et al., 2014). miR-9-3p and miR-151-5p have not been implicated in immune signaling yet. Somewhat unexpected, some of the miRNAs commonly down-regulated in IL10-APCs and DEX-APCs were reported to exert antiinflammatory effects. Thus, miR-130b-3p was shown to directly inhibit the mRNA stability of NF-κB p65, TNF-α, and SP1, and thereby to negatively regulate their expression in hepatocytes (Zheng et al., 2016). Similarly, miR-92a-3p was demonstrated to attenuate TLR-mediated activation of macrophages by inhibiting MKK4 required for JNK activation (Lai et al., 2013). However, our observation of diminished expression of these miRNAs in tolerogenic APC populations suggests that they are not required to promote tolerance.

Besides the group of miRNAs commonly regulated in the differentially tolerized APCs, within the panels of miRNAs specifically upregulated by only one of either tolerizering agent several may exert protolerogenic function as well. Their potential immunogically relevant targets and known functional outcome in immune cells are listed in Table S13.

In a number of studies, stimulation of DCs was shown to strongly alter their transcriptome and proteom, which is substantially regulated by differentially expressed miRNAs (reviewed in Zhou and Wu, 2017). As previously reported (Smyth et al., 2015), mmu-miR-155 was among the miRNAs upregulated in mDC as compared with iDC. In addition, several miRNAs identified in our study as attenuated in expression in unstimulated tolerogenic APCs as compared with iDCs (e.g., mmu-miR-9-5p) were enhanced in expression in mDCs as compared with iDCs. However, besides miRNAs with DC-activating properties like mmu-miR-155-5p, several miRNAs including mmu-miR-34a that were shown to limit the activation of APCs (Kurowska-Stolarska et al., 2017) were found upregulated in mDCs in our study as well. Somewhat surprisingly, we observed that such miRNAs were down-regulated in tolerized APC in response to stimulation with LPS. Further studies are required to elucidate their role in the maintenance of a maturation-resistant tolerogenic state in APCs.

Within the panel of miRNAs convergingly regulated in both tolerogenic, LPS-stimulated APC populations only miRNA-223-3p and miRNA-1224-5p were found upregulated. In contrast to our finding of LPS-mediated down-regulation of mmu-1224-5p in mDC, systemic administration of LPS was reported to enhance its expression in several mouse tissues including spleen (Niu et al., 2011). Overexpression of mmu-1224-5p in a macrophage cell line limited expression of the transcription factor SP1 which in turn diminished TNF-α expression. In contrast to mmu-miR-1224-5p, quite a number of studies have focussed on the functional role of mmu-miR-223-3p. Similar to our findings of LPS-induced down-regulation of mmu-miR-223-3p in mDC, mouse macrophages stimulated with LPS were shown to express this miRNA at attenuated extent (Chen et al., 2012). mmu-miR-223-3p has been thoroughly demonstrated to exert overall antiinflammatory effects in leukocytes in human and mouse (Gaudet et al., 2017; Robertson et al., 2017). Moreover, in human hu-miR-223-3p was identified as a critical modulator of myeloid differentiation (Fazi et al., 2005), and miR-223^−/−^ mice are characterized by diminished granulocytic differentiation and neutrophil activation (Johnnidis et al., 2008). In the course of monocyte/macrophage differentiation, mmu-miR-223 was found to target IKK-α and thereby to inhibit NF-κB activation (Li et al., 2010). In addition, miR-223^−/−^ mice showed elevated numbers of monocyte-derived CD103^+^ DCs, and these generated stronger proinflammatory responses upon stimulation than WT cells (Zhou et al., 2015). Furthermore, mmu-miR-223-3p constitutes a target of PPAR-γ (Ying et al., 2015) and itself targets the transcription factor Pknox1 which negatively regulates polarization of alternative/protolerogenic M2 alternative macrophages (Zhuang et al., 2012). In agreement, mmu-miR-223^−/−^ mice were found to contain much higher levels of inflammatory M1 macrophages. Since a M2-like phenotype and associated protolerogenic function is characteristic of tumor-associated macrophages that promote tumor development, mmu-miR-223 has been attributed an important function in this regard (Szebeni et al., 2017).

Taken together, these studies show that mmu-miR-223-3p plays an important role in the differentiation of leukocytes, and limits the functional activity of differentiated APC populations under homeostatic conditions and in the course of activation. Our finding of convergingly upregulated expression of this miRNA species in differentially tolerized APC populations suggested functional importance of mmu-miRNA-223-3p in this regard. Therefore, we asked for the outcome of transient mmu-miR-223-3p overexpression in BMDC. We show that miR-223 overexpression did not exert a modulatory effect on a panel of other miRNAs identified as differentially regulated in DEX-APCs and IL10-APCs which suggests that their expression is not under control of those transcription factors known to be regulated by miR-223 (Chen et al., 2012, 2016; Agatheeswaran and Chakraborty, 2016). Moreover, mmu-miR-223-3p overexpression in BMDC did not substantially affect the expression of surface markers and cytokines at unstimulated state and after LPS stimulation with the exception of MHCII. However, miR-223 overexpressing BMDCs after stimulation with LPS failed to acquire stimulation-associated T cell stimulatory capacity which may be explained in part by attenuated MHCII expression. In broad accordance with our findings, miR-223^−/−^ Langerhans cells which constitute the epidermal DC population, were shown to mediate much stronger activation of antigen-specific CD8^+^ T cells both *in vitro* and *in vivo* after activation although the expression of activation surface markers was not affected by mmu-miR-223 deficiency (Mi et al., 2013). Altogether, our results show that overexpression of mmu-miR-223-3p in differentiated DCs is sufficient to imprint a maturation-resistant functional state. In order to identify potential targets of this tolerance-promoting miRNA in DCs, we generated a subline of DC-like XS52 cells stably overexpressing miR-223. We identified several validated and predicted mmu-miR-223-3p target mRNAs as down-regulated in this subline. Of these, Kras acts as a cellular signal transmitter that is active when GTP is bound, and is inactivated by hydrolyzation of GTP to GDP due to subsequent conformational changes (Minato, 2013). Rasa1 promotes hydrolysis of RAS-bound GTP to GDP and thereby inhibits Ras activity (King et al., 2013). Rasa1 and Kras commonly regulate RAS signaling and growth factor-responsive signaling pathways which in turn induce MAPK signaling. Cflar has been shown to enhance NF-κB activation (Giampietri et al., 2010). Therefore, our results suggest that mmu-miR-223-3p may establish a protolerogenic state in DCs in part via down-regulation of Rasa1, Kras, and Cflar which in turn affects the activity of several signaling pathways (Ras, MAPK, NF-κB) that contribute to DC activation (Patil et al., 2010).

To the best of our knowledge our study is the first to systematically assess the miRNA signatures of differentially tolerized APCs. In one study, Stumpfova et al. (2014) reported on the miRNA signatures of human monocyte-derived DCs treated with IL-10 plus TGF-ß during maturation, which differed strongly from our results. This discrepancy is most probably due to the fact that we treated mouse bone marrow progenitors with tolerizing agents (DEX, IL-10) on long term under DC-promoting conditions, while in that study differentiated human monocyte-derived DCs were subjected to treatment up to 1 day only. Our finding that transient overexpression of mmu-miR-223-3p was sufficient to imprint a maturation-resistant protolerogenic state in differentiated DCs underscores the suitability of our approach to identify miRNA species as potential targets for immunotherapy (Loyer et al., 2015).

## Conclusion

Differentially tolerized APCs show overlapping miRNA expression patterns, including commonly overexpressed mmu-miR-223-3p. Transient transfection of DC with mmu-miR-223-3p prevented the stimulation-associated acquisition of potent T cell stimulatory capacity. Knowledge of tolerance-associated miRNAs is a prerequisite for the development of immunotherapeutic strategies aimed to reprogram antigen presenting cells.

## Author contributions

MB, MY, VK, DB, FB, E-JS, JS, EM, and SU performed the experiments and analyzed the data. MB, HK, TE, and AR-K designed the study. MB, TE, and AR-K wrote the manuscript.

### Conflict of interest statement

The authors declare that the research was conducted in the absence of any commercial or financial relationships that could be construed as a potential conflict of interest.
