# Supplementary material for: Differentially Tolerized Mouse Antigen Presenting Cells Share a Common miRNA Signature Including Enhanced mmu-miR-223-3p Expression Which Is Sufficient to Imprint a Protolerogenic State
Source: Front Pharmacol. 2018 Aug 17;9:915. doi: 10.3389/fphar.2018.00915 (PMC6108336; doi:10.3389/fphar.2018.00915)
Supplement: Supplementary file 1 [file Data_Sheet_1.doc]

**Supplemental material**

**Table S1.** miRNAs differentially expressed between unstimulated DEX-APCs and iDCs as detected by miRNA array analysis.

| **miRNA** | **Fold Change**  **(log2 vs. iDC)** | **p value** |
| --- | --- | --- |
| mmu-miR-551b-3p | 3.47 | 2.19E-3 |
| mmu-miR-223-3p | 2.59 | 2.19E-3 |
| mmu-miR-30d-5p | 2.15 | 1.37E-2 |
| mmu-miR-27b-3p | 2.03 | 4.37E-2 |
| mmu-miR-23b-3p | 1.84 | 2.89E-2 |
| mmu-miR-30b-5p | 1.84 | 5.39E-3 |
| mmu-miR-365-3p | 1.82 | 1.95E-2 |
| mmu-miR-135a-5p | 1.71 | 1.55E-4 |
| mmu-miR-1895 | 1.64 | 1.95E-2 |
| mmu-miR-16-5p | 1.58 | 6.37E-4 |
| mmu-miR-211-5p | 1.44 | 2.89E-2 |
| mmu-miR-328-3p | 1.40 | 2.55E-2 |
| mmu-miR-1894-5p | 1.40 | 4.35E-2 |
| mmu-miR-709 | 1.36 | 1.54E-2 |
| mmu-let-7d-3p | 1.33 | 2.89E-2 |
| mmu-miR-26b-5p | 1.28 | 1.95E-2 |
| mmu-miR-150-5p | 1.28 | 1.54E-2 |
| mmu-miR-99a-5p | 1.23 | 2.95E-2 |
| mmu-miR-181a-1-3p | 1.23 | 4.46E-2 |
| mmu-let-7b-3p | 1.21 | 4.37E-2 |
| mmu-miR-483-3p | 1.20 | 3.03E-2 |
| mmu-miR-451a | 1.20 | 3.64E-2 |
| mmu-let-7i-3p | 1.18 | 3.60E-2 |
| mmu-miR-142a-5p | -1.34 | 4.37E-2 |
| mmu-miR-425-5p | -1.38 | 5.29E-3 |
| mmu-miR-714 | -1.18 | 4.37E-2 |
| mmu-miR-301b-3p | -1.21 | 3.03E-2 |
| mmu-miR-149-5p | -1.23 | 3.03E-2 |
| mmu-miR-19a-3p | -1.23 | 1.41E-2 |
| mmu-let-7g-5p | -1.23 | 3.88E-2 |
| mmu-miR-712-5p | -1.24 | 1.51E-2 |

| **miRNA** | **Fold Change**  **(log2 vs. iDC)** | **p value** |
| --- | --- | --- |
| mmu-miR-455-5p | -1.24 | 2.37E-2 |
| mmu-miR-301a-3p | -1.25 | 3.03E-2 |
| mmu-miR-33-5p | -1.26 | 4.35E-2 |
| mmu-miR-874-3p | -1.28 | 1.82E-2 |
| mmu-miR-34b-5p | -1.28 | 1.23E-2 |
| mmu-miR-429-3p | -1.28 | 1.10E-2 |
| mmu-miR-20b-5p | -1.29 | 8.54E-3 |
| mmu-miR-1906 | -1.30 | 6.86E-3 |
| mmu-miR-17-3p | -1.34 | 2.89E-2 |
| mmu-miR-877-5p | -1.34 | 2.89E-2 |
| mmu-miR-20a-5p | -1.35 | 1.54E-2 |
| mmu-miR-331-3p | -1.37 | 4.37E-2 |
| mmu-miR-28a-5p | -1.38 | 1.01E-2 |
| mmu-miR-29c-3p | -1.39 | 2.55E-3 |
| mmu-miR-130b-3p | -1.39 | 2.75E-3 |
| mmu-miR-92a-3p | -1.40 | 8.16E-3 |
| mmu-miR-290a-5p | -1.41 | 1.34E-3 |
| mmu-miR-142a-3p | -1.41 | 4.58E-3 |
| mmu-miR-19b-3p | -1.44 | 4.58E-3 |
| mmu-miR-212-3p | -1.46 | 1.51E-3 |
| mmu-miR-200b-3p | -1.49 | 1.58E-3 |
| mmu-miR-34a-5p | -1.51 | 1.34E-3 |
| mmu-miR-674-3p | -1.51 | 8.11E-3 |
| mmu-miR-680 | -1.54 | 1.01E-2 |
| mmu-miR-326-3p | -1.61 | 9.03E-4 |
| mmu-miR-342-3p | -2.11 | 2.44E-3 |
| mmu-miR-151-5p | -2.15 | 5.61E-4 |
| mmu-miR-146a-5p | -2.40 | 8.07E-4 |
| mmu-miR-9-3p | -2.72 | 4.00E-6 |
| mmu-miR-9-5p | -3.77 | 5.00E-6 |
| mmu-miR-132-3p | -4.04 | 8.70E-5 |
| mmu-miR-210-3p | -4.50 | 1.00E-6 |
| mmu-miR-155-5p | -11.63 | 8.00E-6 |

**Table S2.** miRNAs differentially expressed between unstimulated IL10-APCs and iDCs as detected by miRNA arrary analysis.

| **miRNA** | **Fold Change**  **(log2 vs. iDC)** | **p value** |
| --- | --- | --- |
| mmu-miR-494-3p | 1.21 | 1.35E-2 |
| mmu-miR-21a-5p | 1.19 | 1.33E-4 |
| mmu-miR-27b-3p | 1.16 | 9.43E-3 |
| mmu-miR-22-3p | 1.15 | 8.38E-3 |
| mmu-miR-1906 | 1.15 | 2.87E-3 |
| mmu-miR-188-5p | 1.14 | 2.48E-2 |
| mmu-miR-340-5p | 1.12 | 2.48E-2 |
| mmu-miR-24-3p | 1.11 | 2.46E-2 |
| mmu-miR-98-5p | -1.10 | 3.23E-2 |
| mmu-miR-130b-3p | -1.12 | 1.68E-2 |
| mmu-miR-15b-5p | -1.12 | 1.35E-2 |
| mmu-miR-181b-1-3p | -1.12 | 2.99E-3 |
| mmu-miR-92a-3p | -1.16 | 1.33E-3 |
| mmu-miR-146a-5p | -1.22 | 8.38E-3 |
| mmu-miR-151-5p | -1.37 | 4.00E-6 |
| mmu-miR-9-3p | -1.53 | 1.00E-6 |
| mmu-miR-155-5p | -1.60 | 7.00E-6 |
| mmu-miR-9-5p | -1.71 | 1.00E-6 |

**Table S3.** Biological processes predicted to be altered in DEX-APCs versus iDCs due to differential miRNA expression (see Table S1).

| **KEGG pathway** | **#genes** | **#miRNAs** | **p-value** |
| --- | --- | --- | --- |
| **Signal transduction** |  |  |  |
| PI3K-Akt signaling pathway (mmu04151) | 171 | 50 | 1.23E-58 |
| MAPK signaling pathway (mmu04010) | 134 | 51 | 3.05E-54 |
| Wnt signaling pathway (mmu04310) | 87 | 46 | 6.34E-38 |
| T cell receptor signaling pathway (mmu04660) | 67 | 43 | 9.80E-27 |
| TGF-beta signaling pathway (mmu04350) | 50 | 40 | 1.49E-18 |
| HIF-1 signaling pathway (mmu04066) | 57 | 45 | 3.10E-18 |
| mTOR signaling pathway (mmu04150) | 42 | 44 | 4.07E-17 |
| VEGF signaling pathway (mmu04370) | 34 | 40 | 8.77E-14 |
| B cell receptor signaling pathway (mmu04662) | 38 | 44 | 3.65E-12 |
| Hedgehog signaling pathway (mmu04340) | 28 | 36 | 2.01E-11 |
| Jak-STAT signaling pathway (mmu04630) | 68 | 42 | 5.01E-11 |
| Chemokine signaling pathway (mmu04062) | 81 | 45 | 4.18E-6 |
| Phosphatidylinositol signaling system (mmu04070) | 43 | 40 | 2.16E-8 |
| Calcium signaling pathway (mmu04020) | 75 | 48 | 1.14E-6 |
| Fc epsilon RI signaling pathway (mmu04664) | 33 | 39 | 3.12E-6 |
| Notch signaling pathway (mmu04330) | 22 | 30 | 5.74E-5 |
| Toll-like receptor signaling pathway (mmu04620) | 38 | 40 | 4.48E-2 |
| **Cell structure / Motility** | | | |
| Regulation of actin cytoskeleton (mmu04810) | 118 | 52 | 9.53E-50 |
| Focal adhesion (mmu04510) | 117 | 51 | 8.87E-46 |
| Adherens junction (mmu04520) | 44 | 41 | 1.25E-10 |
| Gap junction (mmu04540) | 41 | 42 | 3.43E-10 |
| Leukocyte transendothelial migration (mmu04670) | 56 | 44 | 1.37E-4 |
| **Uptake** | | | |
| Fc gamma R-mediated phagocytosis (mmu04666) | 55 | 42 | 3.92E-22 |
| **Metabolism** | | | |
| Ubiquitin mediated proteolysis (mmu04120) | 80 | 47 | 6.44E-33 |
| Protein processing in endoplasmic reticulum (mmu04141) | 90 | 45 | 6.16E-23 |
| Protein digestion and absorption (mmu04974) | 44 | 34 | 1.08E-17 |
| Apoptosis (mmu04210) | 45 | 39 | 3.85E-14 |
| Inositol phosphate metabolism (mmu00562) | 29 | 37 | 6.49E-6 |
| Sphingolipid metabolism (mmu00600) | 25 | 33 | 1.10E-5 |
| RNA degradation (mmu03018) | 33 | 37 | 5.47E-4 |
| **Metabolism** | | | |
| Glycosphingolipid biosynthesis - ganglio series (mmu00604) | 8 | 12 | 9.50E-4 |
| mRNA surveillance pathway (mmu03015) | 39 | 43 | 7.75E-3 |

**Table S4.** Biological processes predicted to be altered in IL10-APCs versus iDCs due to differential miRNA expression (see Table S2).

| **KEGG pathway** | **#genes** | **#miRNAs** | **p-value** |
| --- | --- | --- | --- |
| **Signal transduction** | | | |
| MAPK signaling pathway (mmu04010) | 104 | 16 | 3.90E-29 |
| PI3K-Akt signaling pathway (mmu04151) | 122 | 16 | 1.48E-23 |
| Wnt signaling pathway (mmu04310) | 67 | 15 | 8.02E-23 |
| T cell receptor signaling pathway (mmu04660) | 49 | 16 | 7.45E-22 |
| TGF-beta signaling pathway (mmu04350) | 37 | 14 | 2.91E-15 |
| Hedgehog signaling pathway (mmu04340) | 24 | 13 | 1.05E-14 |
| mTOR signaling pathway (mmu04150) | 30 | 16 | 7.69E-14 |
| VEGF signaling pathway (mmu04370) | 26 | 15 | 1.47E-8 |
| B cell receptor signaling pathway (mmu04662) | 28 | 15 | 7.79E-7 |
| Fc epsilon RI signaling pathway (mmu04664) | 26 | 15 | 1.46E-5 |
| Chemokine signaling pathway (mmu04062) | 58 | 16 | 2.00E-5 |
| HIF-1 signaling pathway (mmu04066) | 37 | 13 | 1.51E-4 |
| Jak-STAT signaling pathway (mmu04630) | 44 | 16 | 1.62E-3 |
| RIG-I-like receptor signaling pathway (mmu04622) | 20 | 14 | 2.10E-2 |
| Calcium signaling pathway (mmu04020) | 48 | 15 | 2.89E-2 |
| Phosphatidylinositol signaling system (mmu04070) | 26 | 14 | 3.06E-2 |
| **Cell structure / Motility** | | | |
| Regulation of actin cytoskeleton (mmu04810) | 86 | 16 | 4.06E-23 |
| Focal adhesion (mmu04510) | 79 | 16 | 1.99E-22 |
| Adherens junction (mmu04520) | 30 | 13 | 4.52E-5 |
| Gap junction (mmu04540) | 28 | 16 | 4.52E-5 |
| Leukocyte transendothelial migration (mmu04670) | 39 | 14 | 2.72E-3 |
| **Uptake** | | | |
| Fc gamma R-mediated phagocytosis (mmu04666) | 38 | 15 | 2.90E-14 |
| Endocytosis (mmu04144) | 74 | 15 | 1.12E-11 |
| **Metabolism** | | | |
| Ubiquitin mediated proteolysis (mmu04120) | 52 | 17 | 2.51E-10 |
| Protein processing in endoplasmic reticulum (mmu04141) | 62 | 14 | 9.67E-9 |
| Apoptosis (mmu04210) | 28 | 14 | 2.70E-3 |
| Sphingolipid metabolism (mmu00600) | 17 | 10 | 5.88E-3 |
| Glycerophospholipid metabolism (mmu00564) | 31 | 14 | 1.79E-2 |
| Protein digestion and absorption (mmu04974) | 24 | 13 | 2.69E-2 |

**Table S5.** miRNAs differentially expressed between mDCs and iDCs as detected by miRNA array analysis.

| **miRNA** | **Fold Change**  **(log2 vs. iDC)** | **p value** |
| --- | --- | --- |
| mmu-miR-155-5p | 1.80 | 1.00E-6 |
| mmu-miR-29b-3p | 1.33 | 1.00E-6 |
| mmu-miR-29a-3p | 1.31 | 1.00E-6 |
| mmu-miR-34a-5p | 1.28 | 1.00E-6 |
| mmu-miR-146a-5p | 1.26 | 1.10E-5 |
| mmu-miR-455-3p | 1.23 | 1.00E-6 |
| mmu-miR-101b-3p | 1.20 | 1.50E-5 |
| mmu-miR-7a-5p | 1.18 | 1.00E-6 |
| mmu-miR-222-3p | 1.17 | 7.60E-5 |
| mmu-miR-101a-3p | 1.15 | 1.10E-5 |
| mmu-miR-22-5p | 1.14 | 1.13E-4 |
| mmu-miR-9-5p | 1.12 | 1.26E-3 |
| mmu-miR-674-3p | 1.11 | 2.20E-4 |
| mmu-miR-96-5p | 1.11 | 3.33E-4 |
| mmu-miR-193b-3p | 1.11 | 1.09E-3 |
| mmu-miR-9-3p | 1.10 | 1.30E-4 |
| mmu-miR-365-3p | 1.09 | 1.82E-4 |
| mmu-miR-193-5p | 1.09 | 3.14E-3 |
| mmu-miR-22-5p | 1.09 | 1.26E-3 |
| mmu-miR-455-5p | 1.09 | 3.44E-4 |
| mmu-miR-98-5p | 1.09 | 7.52E-4 |
| mmu-miR-147-3p | 1.08 | 2.08E-3 |
| mmu-miR-186-5p | 1.08 | 6.16E-3 |
| mmu-miR-582-5p | 1.07 | 2.50E-2 |
| mmu-miR-29c-3p | 1.07 | 2.50E-2 |
| mmu-miR-92a-3p | 1.07 | 2.18E-3 |
| mmu-miR-674-5p | 1.06 | 1.19E-2 |
| mmu-miR-194-5p | 1.06 | 1.02E-2 |
| mmu-let-7c-5p | 1.06 | 3.81E-2 |
| mmu-miR-26a-5p | 1.06 | 2.30E-2 |
| mmu-miR-20b-5p | 1.06 | 2.86E-2 |
| mmu-miR-361-5p | 1.06 | 1.03E-2 |
| mmu-miR-7a-1-3p | 1.06 | 2.61E-2 |
| **miRNA** | **Fold Change**  **(log2 vs. iDC)** | **p value** |
| mmu-miR-200c-3p | 1.06 | 2.53E-2 |
| mmu-miR-149-5p | 1.05 | 2.51E-2 |
| mmu-miR-141-3p | 1.05 | 3.05E-2 |
| mmu-let-7i-5p | 1.05 | 1.06E-2 |
| mmu-miR-449a-5p | 1.05 | 3.20E-2 |
| mmu-miR-7b-5p | 1.05 | 1.84E-2 |
| mmu-miR-328-3p | 1.05 | 4.51E-2 |
| mmu-miR-130a-3p | 1.05 | 3.20E-2 |
| mmu-miR-345-5p | 1.05 | 2.50E-2 |
| mmu-miR-425-5p | 1.05 | 2.53E-2 |
| mmu-miR-19a-3p | 1.05 | 2.02E-2 |
| mmu-miR-330-3p | 1.04 | 4.97E-2 |
| mmu-miR-27a-3p | -1.19 | 1.00E-6 |
| mmu-miR-1896 | -1.09 | 1.19E-2 |
| mmu-miR-714 | -1.05 | 2.57E-2 |
| mmu-miR-340-3p | -1.05 | 2.18E-2 |
| mmu-miR-140-5p | -1.05 | 3.65E-2 |
| mmu-miR-1901 | -1.06 | 1.69E-2 |
| mmu-miR-188-5p | -1.06 | 2.57E-2 |
| mmu-miR-324-5p | -1.06 | 9.32E-3 |
| mmu-miR-877-5p | -1.06 | 2.54E-2 |
| mmu-miR-874-3p | -1.07 | 2.00E-3 |
| mmu-miR-1906-1 | -1.07 | 1.12E-2 |
| mmu-miR-292-5p | -1.07 | 4.43E-3 |
| mmu-miR-712-5p | -1.07 | 2.80E-3 |
| mmu-miR-221-3p | -1.08 | 1.29E-2 |
| mmu-miR-15b-5p | -1.08 | 1.22E-3 |
| mmu-miR-23b-3p | -1.08 | 1.15E-2 |
| mmu-miR-24-3p | -1.09 | 7.40E-4 |
| mmu-miR-181a-3p | -1.09 | 1.99E-3 |
| mmu-miR-21-5p | -1.09 | 1.26E-3 |
| mmu-miR-342-3p | -1.09 | 7.98E-4 |
| mmu-miR-139-3p | -1.09 | 3.23E-4 |
| mmu-miR-338-3p | -1.11 | 8.50E-3 |
| mmu-miR-1897-5p | -1.11 | 7.90E-5 |
| mmu-miR-378a-5p | -1.14 | 1.78E-3 |
| mmu-miR-27b-3p | -1.14 | 5.00E-5 |
| mmu-miR-721 | -1.14 | 5.00E-6 |
| mmu-miR-290-5p | -1.19 | 1.10E-5 |
| mmu-miR-223-3p | -1.27 | 2.06E-4 |
| mmu-miR-680-1 | -1.30 | 1.00E-6 |
| mmu-miR-210-3p | -1.42 | 1.00E-6 |
| mmu-miR-1224-5p | -1.63 | 1.00E-6 |

**Table S6.** Biological processes predicted to be altered in mDCs versus iDCs due to differential miRNA expression (see Table S5).

| **KEGG pathway** | **#genes** | **#miRNAs** | **p-value** |
| --- | --- | --- | --- |
| **Signal transduction** | | | |
| PI3K-Akt signaling pathway (mmu04151) | 182 | 65 | 1.99E-60 |
| MAPK signaling pathway (mmu04010) | 144 | 64 | 5.64E-49 |
| Wnt signaling pathway (mmu04310) | 93 | 59 | 1.87E-33 |
| Chemokine signaling pathway (mmu04062) | 96 | 61 | 3.24E-26 |
| T cell receptor signaling pathway (mmu04660) | 63 | 59 | 1.80E-21 |
| TGF-beta signaling pathway (mmu04350) | 48 | 52 | 1.45E-18 |
| HIF-1 signaling pathway (mmu04066) | 58 | 56 | 3.02E-17 |
| mTOR signaling pathway (mmu04150) | 43 | 56 | 7.36E-15 |
| VEGF signaling pathway (mmu04370) | 35 | 54 | 4.37E-12 |
| Hedgehog signaling pathway (mmu04340) | 29 | 44 | 4.31E-10 |
| Notch signaling pathway (mmu04330) | 26 | 33 | 4.45E-9 |
| B cell receptor signaling pathway (mmu04662) | 37 | 57 | 3.59E-8 |
| Fc epsilon RI signaling pathway (mmu04664) | 35 | 52 | 2.33E-7 |
| Jak-STAT signaling pathway (mmu04630) | 67 | 55 | 4.31E-7 |
| Calcium signaling pathway (mmu04020) | 79 | 59 | 7.53E-7 |
| Phosphatidylinositol signaling system (mmu04070) | 42 | 48 | 4.72E-6 |
| NOD-like receptor signaling pathway (mmu04621) | 27 | 37 | 2.93E-2 |
| **Cell structure / Motility** | | | |
| Regulation of actin cytoskeleton (mmu04810) | 125 | 64 | 1.95E-43 |
| Focal adhesion (mmu04510) | 118 | 66 | 7.91E-39 |
| Adherens junction (mmu04520) | 48 | 51 | 2.31E-20 |
| Gap junction (mmu04540) | 44 | 60 | 3.84E-17 |
| Tight junction (mmu04530) | 71 | 56 | 8.53E-11 |
| Leukocyte transendothelial migration (mmu04670) | 60 | 56 | 6.41E-6 |
| **Uptake** | | | |
| Endocytosis (mmu04144) | 125 | 59 | 1.82E-44 |
| Fc gamma R-mediated phagocytosis (mmu04666) | 53 | 54 | 4.45E-18 |
| **Metabolism** | | | |
| Ubiquitin mediated proteolysis (mmu04120) | 79 | 62 | 2.16E-27 |
| Protein processing in endoplasmic reticulum (mmu04141) | 94 | 62 | 1.03E-25 |
| Apoptosis (mmu04210) | 41 | 47 | 5.90E-5 |
| Sphingolipid metabolism (mmu00600) | 24 | 36 | 8.16E-4 |
| mRNA surveillance pathway (mmu03015) | 42 | 51 | 1.17E-3 |
| **Metabolism** | | | |
| Citrate cycle (TCA cycle) (mmu00020) | 17 | 19 | 2.69E-3 |
| Inositol phosphate metabolism (mmu00562) | 28 | 41 | 8.69E-3 |

**Table S7.** miRNAs differentially expressed between LPS-stimulated DEX-APCs and mDCs as detected by miRNA array analysis.

| **miRNA** | **Fold Change**  **(log2 vs. iDC)** | **p value** |
| --- | --- | --- |
| mmu-miR-223-3p | 2.77 | 1.00E-6 |
| mmu-miR-1224-5p | 2.44 | 1.00E-6 |
| mmu-miR-551b-3p | 1.56 | 1.00E-6 |
| mmu-miR-135a-5p | 1.47 | 1.00E-6 |
| mmu-miR-15a-5p | 1.40 | 1.00E-6 |
| mmu-miR-1897-5p | 1.37 | 1.00E-6 |
| mmu-miR-146a-5p | 1.34 | 8.00E-6 |
| mmu-miR-340-5p | 1.33 | 1.00E-6 |
| mmu-miR-30d-5p | 1.32 | 1.00E-6 |
| mmu-miR-16-5p | 1.31 | 4.00E-6 |
| mmu-miR-150-5p | 1.29 | 1.00E-6 |
| mmu-miR-340-3p | 1.29 | 1.00E-6 |
| mmu-miR-338-3p | 1.29 | 3.51E-4 |
| mmu-miR-721 | 1.28 | 1.00E-6 |
| mmu-miR-139-5p | 1.26 | 3.00E-6 |
| mmu-miR-1895 | 1.26 | 2.00E-6 |
| mmu-miR-188-5p | 1.25 | 2.00E-6 |
| mmu-miR-31-5p | 1.25 | 3.00E-6 |
| mmu-miR-18a-5p | 1.20 | 2.83E-4 |
| mmu-miR-140-5p | 1.19 | 2.70E-5 |
| mmu-miR-652-3p | 1.17 | 4.80E-5 |
| mmu-miR-93-5p | 1.17 | 1.72E-4 |
| mmu-miR-148a-3p | 1.17 | 2.25E-4 |
| mmu-miR-574-5p | 1.15 | 1.29E-4 |
| mmu-miR-126-3p | 1.13 | 3.74E-3 |
| mmu-miR-31-3p | 1.13 | 5.60E-4 |
| mmu-miR-680-1 | 1.12 | 1.56E-3 |
| mmu-miR-26b-5p | 1.11 | 1.09E-2 |
| mmu-miR-30e-5p | 1.11 | 2.47E-3 |
| mmu-miR-10a-5p | 1.10 | 1.09E-2 |
| mmu-miR-451a | 1.10 | 6.75E-3 |
| mmu-miR-1904 | 1.09 | 6.75E-3 |
| mmu-miR-27a-3p | 1.09 | 6.75E-3 |
| **miRNA** | **Fold Change**  **(log2 vs. iDC)** | **p value** |
| mmu-miR-1894-3p | 1.09 | 5.03E-3 |
| mmu-miR-30c-5p | 1.09 | 1.70E-2 |
| mmu-miR-181a-1-3p | 1.09 | 1.42E-2 |
| mmu-miR-365-3p | 1.09 | 2.84E-2 |
| mmu-miR-362-5p | 1.09 | 2.88E-2 |
| mmu-miR-1896 | 1.09 | 1.25E-2 |
| mmu-miR-1187 | 1.08 | 2.26E-2 |
| mmu-miR-30a-5p | 1.07 | 4.19E-2 |
| mmu-miR-19a-3p | 1.07 | 2.96E-2 |
| mmu-miR-208a-3p | 1.07 | 2.72E-2 |
| mmu-miR-1892 | 1.07 | 2.44E-2 |
| mmu-let-7b-3p | 1.06 | 4.60E-2 |
| mmu-miR-107-3p | 0.83 | 2.17E-4 |
| mmu-miR-483-5p | 1.39 | 5.00E-6 |
| mmu-miR-30b-5p | 1.22 | 1.51E-4 |
| mmu-miR-140-3p | 1.21 | 5.10E-5 |
| mmu-miR-25-3p | 1.18 | 9.90E-5 |
| mmu-miR-15b-5p | -1.18 | 7.50E-5 |
| mmu-miR-675-5p | 1.08 | 4.21E-2 |
| mmu-miR-20a-3p | -1.08 | 1.23E-2 |
| mmu-miR-186-5p | -1.11 | 2.84E-3 |
| mmu-miR-185-5p | -1.10 | 3.74E-3 |
| mmu-let-7a-5p | -1.11 | 1.22E-3 |
| mmu-miR-29a-3p | -1.52 | 1.00E-6 |
| mmu-miR-29b-3p | -1.86 | 1.00E-6 |
| mmu-miR-132-3p | -1.96 | 1.00E-6 |
| mmu-miR-1191a | -1.06 | 4.34E-2 |
| mmu-miR-130b-5p | -1.06 | 4.93E-2 |
| mmu-miR-324-5p | -1.07 | 4.00E-2 |
| mmu-miR-429-3p | -1.07 | 3.68E-2 |
| mmu-miR-128-3p | -1.07 | 3.79E-2 |
| mmu-let-7g-5p | -1.08 | 1.48E-2 |
| mmu-miR-22-3p | -1.09 | 3.03E-2 |
| mmu-miR-28a-5p | -1.09 | 1.19E-2 |
| mmu-let-7i-5p | -1.09 | 8.14E-3 |
| mmu-miR-505-3p | -1.09 | 3.98E-2 |
| mmu-let-7c-5p | -1.09 | 3.96E-2 |
| mmu-miR-29a-5p | -1.09 | 1.23E-2 |
| mmu-miR-200a-3p | -1.09 | 8.57E-3 |
| mmu-miR-26a-5p | -1.09 | 7.87E-3 |
| mmu-miR-29b-1-5p | -1.09 | 3.96E-3 |
| mmu-miR-669f-3p | -1.10 | 1.23E-2 |
| mmu-miR-342-5p | -1.11 | 2.17E-3 |
| mmu-miR-455-5p | -1.12 | 4.07E-3 |
| mmu-miR-32-5p | -1.13 | 1.41E-3 |
| mmu-miR-669a-1 | -1.13 | 1.53E-3 |
| mmu-miR-149-5p | -1.13 | 6.89E-4 |
| mmu-miR-425-5p | -1.13 | 6.89E-4 |
| mmu-miR-147-3p | -1.13 | 6.89E-4 |
| mmu-miR-200b-3p | -1.14 | 1.49E-3 |
| mmu-miR-674-5p | -1.14 | 5.69E-4 |
| mmu-miR-96-5p | -1.14 | 2.47E-3 |
| mmu-miR-193b-3p | -1.15 | 4.35E-4 |
| mmu-miR-142-3p | -1.17 | 4.69E-4 |
| mmu-miR-449a-5p | -1.17 | 7.00E-5 |
| mmu-miR-210-3p | -1.17 | 2.72E-2 |
| mmu-miR-690 | -1.18 | 9.09E-3 |
| mmu-miR-29c-3p | -1.18 | 8.43E-4 |
| mmu-miR-34c-5p | -1.18 | 2.10E-5 |
| mmu-miR-500-3p | -1.18 | 1.22E-2 |
| mmu-miR-194-5p | -1.18 | 2.90E-5 |
| mmu-miR-212-3p | -1.19 | 3.60E-5 |
| mmu-miR-350-3p | -1.20 | 9.34E-4 |
| mmu-miR-22-5p | -1.21 | 1.56E-4 |
| mmu-miR-101b-3p | -1.21 | 8.00E-6 |
| mmu-let-7f-5p | -1.22 | 1.60E-5 |
| mmu-miR-34b-5p | -1.22 | 8.00E-6 |
| mmu-miR-331-3p | -1.23 | 4.00E-6 |
| mmu-miR-221-3p | -1.23 | 9.00E-5 |
| mmu-miR-101a-3p | -1.24 | 5.00E-6 |
| mmu-miR-146b-5p | -1.25 | 5.60E-4 |
| mmu-miR-98-5p | -1.25 | 4.00E-6 |
| mmu-miR-7a-5p | -1.26 | 2.00E-6 |
| mmu-miR-326-3p | -1.27 | 1.60E-5 |
| mmu-let-7d-5p | -1.27 | 4.00E-6 |
| mmu-miR-222-3p | -1.29 | 4.00E-6 |
| mmu-miR-455-5p | -1.38 | 1.00E-6 |
| mmu-miR-674-3p | -1.42 | 1.00E-6 |
| mmu-miR-151-5p | -1.56 | 1.00E-6 |
| mmu-miR-34a-5p | -1.57 | 1.00E-6 |
| mmu-miR-342-3p | -1.66 | 1.00E-6 |
| mmu-miR-9-3p | -1.72 | 1.00E-6 |
| mmu-miR-9-5p | -2.00 | 1.00E-6 |
| mmu-miR-155-5p | -3.35 | 1.00E-6 |

**Table S8.** miRNAs differentially expressed between LPS-stimulated IL10-APCs and mDCs as detected by miRNA array analysis.

| **miRNA** | **Fold Change**  **(log2 vs. iDC)** | **p value** |
| --- | --- | --- |
| mmu-miR-1224-5p | 4.45 | 4.45E-2 |
| mmu-miR-223-3p | 3.43 | 4.45E-2 |
| mmu-miR-21a-5p | 2.09 | 4.45E-2 |
| mmu-miR-455-3p | -1.80 | 4.19E-2 |
| mmu-miR-34a-5p | -1.98 | 4.19E-2 |
| mmu-miR-29a-3p | -2.11 | 4.19E-2 |
| mmu-miR-9-3p | -3.45 | 4.45E-2 |
| mmu-miR-155-5p | -3.56 | 1.52E-2 |
| mmu-miR-9-5p | -4.89 | 4.19E-2 |

**Table S9.** Biological processes predicted to be altered in stimulated DEX-APCs versus mDCs due to differential miRNA expression (see Table S7).

| **KEGG pathway** | **#genes** | **#miRNAs** | **p-value** |
| --- | --- | --- | --- |
| **Signal transduction** |  |  |  |
| PI3K-Akt signaling pathway (mmu04151) | 264 | 96 | 4.94E-38 |
| MAPK signaling pathway (mmu04010) | 163 | 99 | 1.93E-31 |
| Wnt signaling pathway (mmu04310) | 111 | 94 | 2.48E-23 |
| Calcium signaling pathway (mmu04020) | 102 | 93 | 3.35E-20 |
| Chemokine signaling pathway (mmu04062) | 103 | 91 | 4.77E-20 |
| Jak-STAT signaling pathway (mmu04630) | 81 | 89 | 6.32E-16 |
| T cell receptor signaling pathway (mmu04660) | 74 | 91 | 7.26E-15 |
| Phosphatidylinositol signaling system (mmu04070) | 52 | 82 | 2.65E-13 |
| TGF-beta signaling pathway (mmu04350) | 56 | 77 | 1.10E-12 |
| HIF-1 signaling pathway (mmu04066) | 62 | 83 | 1.10E-12 |
| mTOR signaling pathway (mmu04150) | 46 | 84 | 1.00E-09 |
| B cell receptor signaling pathway (mmu04662) | 45 | 90 | 1.56E-09 |
| VEGF signaling pathway (mmu04370) | 42 | 85 | 9.00E-09 |
| Fc epsilon RI signaling pathway (mmu04664) | 40 | 80 | 2.08E-08 |
| Hedgehog signaling pathway (mmu04340) | 34 | 70 | 3.19E-07 |
| Notch signaling pathway (mmu04330) | 28 | 62 | 4.80E-06 |
| Basal transcription factors (mmu03022) | 23 | 56 | 4.48E-05 |
| RIG-I-like receptor signaling pathway (mmu04622) | 32 | 66 | 2.71E-03 |
| Citrate cycle (TCA cycle) (mmu00020) | 18 | 27 | 5.08E-03 |
| **Cell structure / Motility** | | | |
| Regulation of actin cytoskeleton (mmu04810) | 140 | 97 | 7.03E-08 |
| Cytokine-cytokine receptor interaction (mmu04060) | 129 | 87 | 1.08E-08 |
| Leukocyte transendothelial migration (mmu04670) | 67 | 79 | 2.73E-05 |
| Focal adhesion (mmu04510) | 136 | 94 | 8.86E-26 |
| Adherens junction (mmu04520) | 55 | 84 | 2.26E-13 |
| **Uptake** | | | |
| Endocytosis (mmu04144) | 137 | 91 | 7.20E-28 |
| Fc gamma R-mediated phagocytosis (mmu04666) | 59 | 83 | 4.86E-12 |
| Gap junction (mmu04540) | 52 | 85 | 7.46E-12 |
| **Metabolism** | | | |
| Protein processing in endoplasmic reticulum (mmu04141) | 103 | 86 | 3.55E-22 |
| Ubiquitin mediated proteolysis (mmu04120) | 88 | 92 | 7.74E-18 |
| Apoptosis (mmu04210) | 53 | 79 | 1.44E-11 |
| **Metabolism** | | | |
| Protein digestion and absorption (mmu04974) | 52 | 73 | 1.24E-10 |
| Sphingolipid metabolism (mmu00600) | 28 | 55 | 1.01E-07 |
| Glycosphingolipid biosynthesis - ganglio series (mmu00604) | 10 | 19 | 2.86E-05 |
| mRNA surveillance pathway (mmu03015) | 46 | 78 | 2.57E-03 |

**Table S10.** Biological processes predicted to be altered in stimulated IL10-APCs versus mDCs due to differential miRNA expression (see Table S8).

| **KEGG pathway** | **#genes** | **#miRNAs** | **p-value** |
| --- | --- | --- | --- |
| **Signal transduction** | | | |
| PI3K-Akt signaling pathway (mmu04151) | 75 | 8 | 1.42E-20 |
| mTOR signaling pathway (mmu04150) | 20 | 8 | 9.52E-11 |
| MAPK signaling pathway (mmu04010) | 50 | 7 | 3.77E-09 |
| T cell receptor signaling pathway (mmu04660) | 25 | 7 | 1.07E-07 |
| B cell receptor signaling pathway (mmu04662) | 16 | 7 | 1.49E-04 |
| Chemokine signaling pathway (mmu04062) | 32 | 7 | 2.71E-04 |
| Notch signaling pathway (mmu04330) | 11 | 4 | 5.62E-04 |
| HIF-1 signaling pathway (mmu04066) | 20 | 7 | 9.39E-04 |
| VEGF signaling pathway (mmu04370) | 13 | 7 | 1.57E-03 |
| Fc epsilon RI signaling pathway (mmu04664) | 13 | 7 | 1.18E-02 |
| **Cell structure / Motility** | | | |
| Focal adhesion (mmu04510) | 52 | 8 | 1.42E-20 |
| ECM-receptor interaction (mmu04512) | 22 | 4 | 6.40E-14 |
| Regulation of actin cytoskeleton (mmu04810) | 46 | 7 | 3.40E-10 |
| Adherens junction (mmu04520) | 18 | 7 | 1.14E-04 |
| Gap junction (mmu04540) | 16 | 7 | 1.37E-03 |
| Tight junction (mmu04530) | 25 | 7 | 1.05E-02 |
| **Uptake** | | | |
| Fc gamma R-mediated phagocytosis (mmu04666) | 19 | 8 | 1.55E-05 |
| Endocytosis (mmu04144) | 34 | 7 | 1.66E-03 |
| **Metabolism** | | | |
| Protein digestion and absorption (mmu04974) | 22 | 5 | 3.68E-09 |
| Ubiquitin mediated proteolysis (mmu04120) | 25 | 8 | 7.01E-04 |
| Protein processing in endoplasmic reticulum (mmu04141) | 29 | 8 | 4.15E-03 |

**Table S11**. mRNAs differentially expressed in XS52 cells engineered to overexpress mmu-miR-223-3p as compared with expression in the corresponding control subline. mRNA expression levels were assessed by array analysis in 2 independent experiments.

| **Gene^1^** | **Description** | **Fold change** |
| --- | --- | --- |
| Tas2r115 | taste receptor. type 2. member 115 | 129.562 |
| Pla2g2a | phospholipase A2. group IIA (platelets. synovial fluid) | 100.900 |
| Kcna6 | potassium voltage-gated channel. shaker-related. subfamily. member 6 | 40.601 |
| Awat1 | acyl-CoA wax alcohol acyltransferase 1 | 40.391 |
| LOC674195|Usp48 | ubiquitin carboxyl-terminal hydrolase 48-like|ubiquitin specific peptidase 48 | 34.630 |
| Tia1 | cytotoxic granule-associated RNA binding protein 1 | 25.950 |
| Gm10825 | predicted gene 10825 | 25.776 |
| Ttc9c | tetratricopeptide repeat domain 9C | 22.606 |
| Zbtb42 | zinc finger and BTB domain containing 42 | 20.935 |
| Gas2l2 | growth arrest-specific 2 like 2 | 20.724 |
| Cyb561d2 | cytochrome b-561 domain containing 2 | 20.330 |
| Nkx2-5 | NK2 transcription factor related. locus 5 (Drosophila) | 18.914 |
| Brd2 | bromodomain containing 2 | 17.426 |
| Nr1d1 | nuclear receptor subfamily 1. group D. member 1 | 16.719 |
| Neu2 | neuraminidase 2 | 15.348 |
| Pan3 | PAN3 polyA specific ribonuclease subunit homolog (S. cerevisiae) | 14.1545 |
| Tnni3k | TNNI3 interacting kinase | 13.396 |
| Zfp651 | zinc finger protein 651 | 13.266 |
| Fcgr2b | Fc receptor. IgG. low affinity IIb | 12.327 |
| Snrnp48 | small nuclear ribonucleoprotein 48 (U11/U12) | 12.0812 |
| Loh12cr1 | loss of heterozygosity. 12. chromosomal region 1 homolog (human) | 11.626 |
| Fbxo30 | F-box protein 30 | 11.502 |
| Ng23 | Ng23 protein | 11.348 |
| Btbd3 | BTB (POZ) domain containing 3 | 11.3103 |
| Ces5a | carboxylesterase 5A | 11.209 |
| Pomgnt1 | protein O-linked mannose beta1.2-N-acetylglucosaminyltransferase | 10.495 |
| Selk | selenoprotein K | 10.460 |
| Ubtd1 | ubiquitin domain containing 1 | 9.570 |
| Aatk | apoptosis-associated tyrosine kinase | 9.501 |
| Ccdc134 | coiled-coil domain containing 134 | 9.316 |
| Gm10636 | predicted gene 10636 | 9.170 |

| **Gene^1^** | **Description** | **Fold change** |
| --- | --- | --- |
| Qk | quaking | 9.057 |
| Gulp1 | GULP. engulfment adaptor PTB domain containing 1 | 9.050 |
| Gm2695 | predicted gene 2695 | 8.574 |
| Nkapl | NFKB activating protein-like | 8.147 |
| Jak3 | Janus kinase 3 | 7.871 |
| Ammecr1 | Alport syndrome. mental retardation. midface hypoplasia and elliptocytosis chromosomal region gene 1 homolog (human) | 7.748 |
| Ccny | cyclin Y | 7.735 |
| Ccdc138 | coiled-coil domain containing 138 | 7.529 |
| Chpt1 | choline phosphotransferase 1 | 7.460 |
| Acvr2b | activin receptor IIB | 7.292 |
| Olfr984 | olfactory receptor 984 | 7.254 |
| Tmc4 | transmembrane channel-like gene family 4 | 7.195 |
| Mir411 | microRNA 411 | 6.947 |
| BC031781 | cDNA sequence BC031781 | 6.855 |
| Nhsl2 | NHS-like 2 | 6.822 |
| Rreb1 | ras responsive element binding protein 1 | 6.651 |
| Upb1 | ureidopropionase. beta | 6.635 |
| Cnot6l | CCR4-NOT transcription complex. subunit 6-like | 6.562 |
| C030039L03Rik | RIKEN cDNA C030039L03 gene | 6.442 |
| Dsg1a | desmoglein 1 alpha | 6.386 |
| LOC100505102|Mcrs1 | microspherule protein 1-like|microspherule protein 1 | 6.203 |
| Zdhhc1 | zinc finger. DHHC domain containing 1 | 6.041 |
| Thtpa | thiamine triphosphatase | 5.917 |
| Pou5f2 | POU domain class 5. transcription factor 2 | 5.803 |
| 1700011F14Rik | RIKEN cDNA 1700011F14 gene | 5.722 |
| Tbcb | tubulin folding cofactor B | 5.653 |
| Spata9 | spermatogenesis associated 9 | 5.591 |
| 1810063B07Rik | RIKEN cDNA 1810063B07 gene | 5.519 |
| C1qtnf4 | C1q and tumor necrosis factor related protein 4 | 5.229 |
| Ndufv3 | NADH dehydrogenase (ubiquinone) flavoprotein 3 | 5.190 |
| Prss36 | protease. serine. 36 | 5.103 |
| Ugt1a9|Ugt1a7c|Ugt1a1|Ugt1a6a|Ugt1a2|Ugt1a10|Ugt1a5 | UDP glucuronosyltransferase 1 family. polypeptide A9|UDP glucuronosyltransferase 1 family. polypeptide A7C|UDP glucuronosyltransferase 1 family. polypeptide A1|UDP glucuronosyltransferase 1 family. polypeptide A6A|UDP glucuronosyltransferase 1 family. pol | 5.082 |
| Dio3 | deiodinase. iodothyronine type III | 5.071 |
| Mogat1 | monoacylglycerol O-acyltransferase 1 | 4.978 |
| Mfsd4 | major facilitator superfamily domain containing 4 | 4.959 |
| 6030405A18Rik | RIKEN cDNA 6030405A18 gene | 4.951 |
| Olfr1317 | olfactory receptor 1317 | 4.903 |
| 4930449E01Rik | RIKEN cDNA 4930449E01 gene | 4.755 |
| Tirap | toll-interleukin 1 receptor (TIR) domain-containing adaptor protein | 4.716 |
| 0610009B22Rik | RIKEN cDNA 0610009B22 gene | 4.465 |
| Gtf2h4 | general transcription factor II H. polypeptide 4 | 4.389 |
| Lce3a | late cornified envelope 3A | 4.336 |
| Gm8005 | predicted gene 8005 | 4.192 |
| Tlr1 | toll-like receptor 1 | 4.188 |
| Pvrl3 | poliovirus receptor-related 3 | 4.048 |
| Il21r | interleukin 21 receptor | 3.940 |
| C2 | complement component 2 (within H-2S) | 3.939 |
| Rnf167 | ring finger protein 167 | 3.909 |
| Cyba | cytochrome b-245. alpha polypeptide | 3.785 |
| Gnat1 | guanine nucleotide binding protein. alpha transducing 1 | 3.757 |
| Krtap22-2 | keratin associated protein 22-2 | 3.707 |
| Cmtm3 | CKLF-like MARVEL transmembrane domain containing 3 | 0.272 |
| Cox6c | cytochrome c oxidase. subunit VIc | 0.271 |
| Hmcn1 | hemicentin 1 | 0.261 |
| Ccrn4l | CCR4 carbon catabolite repression 4-like (S. cerevisiae) | 0.257 |
| Wdr76 | WD repeat domain 76 | 0.255 |
| A530016L24Rik | RIKEN cDNA A530016L24 gene | 0.244 |
| Wnt5a | wingless-related MMTV integration site 5A | 0.240 |
| Ube2z | ubiquitin-conjugating enzyme E2Z (putative) | 0.237 |
| Pacsin2 | protein kinase C and casein kinase substrate in neurons 2 | 0.237 |
| Impact | imprinted and ancient | 0.234 |
| Kcnj4 | potassium inwardly-rectifying channel. subfamily J. member 4 | 0.232 |
| Kras | v-Ki-ras2 Kirsten rat sarcoma viral oncogene homolog | 0.227 |
| Tmem200c | transmembrane protein 200C | 0.224 |
| Rn4.5s | 4.5S RNA | 0.206 |
| Lars | leucyl-tRNA synthetase | 0.206 |
| Clic1 | chloride intracellular channel 1 | 0.203 |
| AK129341 | cDNA sequence AK129341 | 0.202 |
| Serpinb2 | serine (or cysteine) peptidase inhibitor. clade B. member 2 | 0.199 |
| Baz2a | bromodomain adjacent to zinc finger domain. 2A | 0.199 |
| Ucn2 | urocortin 2 | 0.193 |
| 6330503K22Rik | RIKEN cDNA 6330503K22 gene | 0.191 |
| Scd4 | stearoyl-coenzyme A desaturase 4 | 0.178 |
| Rxfp2 | relaxin/insulin-like family peptide receptor 2 | 0.173 |
| Acer2 | alkaline ceramidase 2 | 0.172 |
| Sh3bp4 | SH3-domain binding protein 4 | 0.170 |
| Mir10a | microRNA 10a | 0.168 |
| Camk4 | calcium/calmodulin-dependent protein kinase IV | 0.168 |
| LOC631217 | hypothetical protein LOC631217 | 0.166 |
| Fbxl20 | F-box and leucine-rich repeat protein 20 | 0.160 |
| Gm749 | predicted gene 749 | 0.158 |
| Rasa1 | RAS p21 protein activator 1 | 0.151 |
| 4933400A11Rik | capping protein (actin filament) muscle Z-line. alpha 1 pseudogene | 0.147 |
| Lsm2 | LSM2 homolog. U6 small nuclear RNA associated (S. cerevisiae) | 0.147 |
| Myl10 | myosin. light chain 10. regulatory | 0.145 |
| 4833403I15Rik | RIKEN cDNA 4833403I15 gene | 0.144 |
| Nrbp2 | nuclear receptor binding protein 2 | 0.139 |
| Reg3d | regenerating islet-derived 3 delta | 0.137 |
| E030030I06Rik | RIKEN cDNA E030030I06 gene | 0.135 |
| Mir598 | microRNA 598 | 0.128 |
| Ptpn4 | protein tyrosine phosphatase. non-receptor type 4 | 0.122 |
| Ltbp2 | latent transforming growth factor beta binding protein 2 | 0.121 |
| Zbtb24 | zinc finger and BTB domain containing 24 | 0.119 |
| Sgsh | N-sulfoglucosamine sulfohydrolase (sulfamidase) | 0.119 |
| Fbln1 | fibulin 1 | 0.115 |
| Cflar | CASP8 and FADD-like apoptosis regulator | 0.114 |
| Vps8 | vacuolar protein sorting 8 homolog (S. cerevisiae) | 0.114 |
| Prap1 | proline-rich acidic protein 1 | 0.113 |
| Trmt2b | TRM2 tRNA methyltransferase 2 homolog B (S. cerevisiae) | 0.106 |
| Enpp5 | ectonucleotide pyrophosphatase/phosphodiesterase 5 | 0.103 |
| Ndufa9 | NADH dehydrogenase (ubiquinone) 1 alpha subcomplex. 9 | 0.102 |
| Ttc21a | tetratricopeptide repeat domain 21A | 0.102 |
| Plin4 | perilipin 4 | 0.100 |
| Trappc6a | trafficking protein particle complex 6A | 0.093 |
| Rbm20 | RNA binding motif protein 20 | 0.093 |
| Glt6d1 | glycosyltransferase 6 domain containing 1 | 0.089 |
| Gpr85 | G protein-coupled receptor 85 | 0.085 |
| Mc5r | melanocortin 5 receptor | 0.085 |
| Ptchd1 | patched domain containing 1 | 0.080 |
| Zcchc5 | zinc finger. CCHC domain containing 5 | 0.077 |
| Rad18 | RAD18 homolog (S. cerevisiae) | 0.073 |
| Il17rc | interleukin 17 receptor C | 0.068 |
| Pck2 | phosphoenolpyruvate carboxykinase 2 (mitochondrial) | 0.064 |
| Fto | fat mass and obesity associated | 0.055 |
| Rhox3-ps | reproductive homeobox 3. pseudogene | 0.051 |
| Med4 | mediator of RNA polymerase II transcription. subunit 4 homolog (yeast) | 0.046 |
| Spg20 | spastic paraplegia 20. spartin (Troyer syndrome) homolog (human) | 0.045 |
| 1700019M22Rik|Gm9059 | RIKEN cDNA 1700019M22 gene|predicted gene 9059 | 0.043 |
| Tnf | tumor necrosis factor | 0.035 |
| Ooep | oocyte expressed protein homolog (dog) | 0.032 |
| Birc5 | baculoviral IAP repeat-containing 5 | 0.029 |
| Morc1 | microrchidia 1 | 0.028 |
| B230218P12Rik | RIKEN cDNA B230218P12 gene | 0.027 |
| Mrgprb1 | MAS-related GPR. member B1 | 0.027 |
| Vmn1r195 | vomeronasal 1 receptor 195 | 0.017 |
| Svs1 | seminal vesicle secretory protein 1 | 0.015 |
| Pabpc1 | poly(A) binding protein. cytoplasmic 1 | 0.013 |
| Wdr5 | WD repeat domain 5 | 0.013 |
| Nadsyn1 | NAD synthetase 1 | 0.011 |
| Spib | Spi-B transcription factor (Spi-1/PU.1 related) | 0.010 |
| LOC100504952|Bpnt1 | hypothetical LOC100504952|bisphosphate 3'-nucleotidase 1 | 0.008 |
| Myo1g | myosin IG | 0.007 |
| Per3 | period homolog 3 (Drosophila) | 0.006 |
| Hoxa1 | homeobox A1 | 0.005 |
| Pdk4 | pyruvate dehydrogenase kinase. isoenzyme 4 | 0.004 |
| Tmed4 | transmembrane emp24 protein transport domain containing 4 | 0.003 |
| Piwil1 | piwi-like homolog 1 (Drosophila) | 0.003 |
| Slc38a7 | solute carrier family 38. member 7 | 0.002 |

**^1^** Validated mmu-miR-223-3p targets are indicated by a grey backgroiund.

**Table S12.** Biological processes predicted to be altered in DCs in response to overexpression of mmu-miR-223-3p, and involvement of down-regulated mmu-miR-223-3p targets (see Figure 4).

| **Biological process** | **# of genes** | **including** | | |
| --- | --- | --- | --- | --- |
|  |  | **Cflar** | **Kras** | **Rasa1** |
| Cellular process (GO:0009987) | 65 | ● |  |  |
| Metabolic process (GO:0008152) | 56 |  |  |  |
| Response to stimulus (GO:0050896) | 28 | ● |  | ● |
| Developmental process (GO:0032502) | 16 | ● |  |  |
| Biological regulation (GO:0065007) | 15 | ● |  |  |
| Multicellular organismal process (GO:0032501) | 14 |  | ● |  |
| Localization (GO:0051179) | 12 |  | ● |  |
| Cellular component organization or biogenesis (GO:0071840) | 9 |  |  |  |
| Immune system process (GO:0002376) | 6 |  |  |  |
| Biological adhesion (GO:0022610) | 4 |  | ● |  |
| Locomotion (GO:0040011) | 1 |  |  |  |

**Table S13.** mRNA targets of miRNAs upregulated non-convergingly by DEX-APCs and IL10-APCs at either state of stimulation with relevance for APC activity.

| **miRNA** | **Target mRNA** | **Affected pathway^1^** | **Cell type^2^** | **Species^3^** | **Reference** |
| --- | --- | --- | --- | --- | --- |
| **miR-10a-5p** | Nod2 | NOD receptor signaling, MAPK signaling, NF-κB signaling, cytokine signaling in immune system, innate immune system | monocyte-derived DC | h | Wu W, He C, Liu C, Cao AT, Xue X, Evans-Marin HL, Sun M, Fang L, Yao S, Pinchuk IV, Powell DW, Liu Z, Cong Y. miR-10a inhibits dendritic cell activation and Th1/Th17 cell immune responses in IBD. Gut. 2015;64(11):1755-64. doi: 10.1136/gutjnl-2014-307980 |
|  | IL-12/IL-23p40 | MAPK signaling, TGF-β/Smad signaling, JAK/STAT signaling |  |  |  |
|  | Gata6 | NF-κB signaling | endothelial cells | h | Lee DY, Lin TE, Lee CI, Zhou J, Huang YH, Lee PL, Shih YT, Chien S, Chiu JJ. MicroRNA-10a is crucial for endothelial response to different flow patterns via interaction of retinoid acid receptors and histone deacetylases. Proc Natl Acad Sci U S A. 2017;114(8):2072-2077. doi: 10.1073/pnas.1621425114 |
|  | Klf4 | WNT signaling, NOTCH signaling, DC developmental lineage pathway | MSC | h | Li J, Dong J, Zhang ZH, Zhang DC, You XY, Zhong Y, Chen MS, Liu SM. miR-10a restores human mesenchymal stem cell differentiation by repressing KLF4. J Cell Physiol. 2013;228(12):2324-36. doi: 10.1002/jcp.24402 |
|  | Pik3ca | Akt signaling, MAPK signaling, mTOR signaling | ASMC | h | Hu R, Pan W, Fedulov AV, Jester W, Jones MR, Weiss ST, Panettieri RA Jr, Tantisira K, Lu Q. MicroRNA-10a controls airway smooth muscle cell proliferation via direct targeting of the PI3 kinase pathway. FASEB J. 2014;28(5):2347-57. doi: 10.1096/fj.13-247247 |

| **miRNA** | **Target mRNA** | **Affected pathway^1^** | **Cell type^2^** | **Species^3^** | **Reference** |
| --- | --- | --- | --- | --- | --- |
| **miR-10a-5p** | MAPK8IP1 | MAPK signaling, Akt signaling, TGF-β signaling | GC | h | Lu Y, Wei G, Liu L, Mo Y, Chen Q, Xu L, Liao R, Zeng D, Zhang K. Direct targeting of MAPK8IP1 by miR-10a-5p is a major mechanism for gastric cancer metastasis. Oncol Lett. 2017;13(3):1131-1136. doi: 10.3892/ol.2016.5544 |
| **miR-15a-5p** | Cxcl10 | MAPK signaling, RIG-I/MDA5 mediated induction of IFN type 1 pathways, TLR signaling | PBMC | h | Liu XF, Wang RQ, Hu B, Luo MC, Zeng QM, Zhou H, Huang K, Dong XH, Luo YB, Luo ZH, Yang H. 2015. MiR-15a contributes abnormal immune response in myasthenia gravis by targeting CXCL10. Clin Immunol. 2016;164:106-13. doi: 10.1016/j.clim.2015.12.009 |
|  | PU.1 | NF-κB Signaling, development of DC and macrophage subsets, glucocorticoid receptor regulatory network, | bone marrow-derived macrophages | m | Moon HG, Yang J, Zheng Y, Jin Y. miR-15a/16 regulates macrophage phagocytosis after bacterial infection. J Immunol. 2014;193(9):4558-67. doi: 10.4049/jimmunol.1401372 |
| **miR-18a-5p** | Cdc42 | cytoskeletal signaling, Ras pathway, MAPK signaling, Rho family GTPases, non-canonical WNT signaling | CRC cells | h | Humphreys KJ, McKinnon RA, Michael MZ. miR-18a inhibits CDC42 and plays a tumour suppressor role in colorectal cancer cells. PLoS One. 2014;9(11):e112288. doi: 10.1371/journal.pone.0112288 |
|  | Pias3 | JAK/STAT signaling | gastric tissue | h | Wu W, Takanashi M, Borjigin N, Ohno SI, Fujita K, Hoshino S, Osaka Y, Tsuchida A, Kuroda M. MicroRNA-18a modulates STAT3 activity through negative regulation of PIAS3 during gastric adenocarcinogenesis. Br J Cancer. 2013;108(3):653-61. doi: 10.1038/bjc.2012.587 |
|  | Runx1 | NF-κB Signaling, TGF-β/Smad signaling | K562 cell line | h | Ben-Ami O, Pencovich N, Lotem J, Levanon D, Groner Y. A regulatory interplay between miR-27a and Runx1 during megakaryopoiesis. Proc Natl Acad Sci U S A. 2009;106(1):238-43. doi: 10.1073/pnas.0811466106 |
| **miR-19a-3p** | Socs3 | JAK/STAT signaling, NF-κB signaling, Class I MHC mediated antigen processing and presentation | pancreatic ß cells | h | Li Y, Luo T, Wang L, Wu J, Guo S. MicroRNA-19a-3p enhances the proliferation and insulin secretion, while it inhibits the apoptosis of pancreatic β cells via the inhibition of SOCS3. Int J Mol Med. 2016;38(5):1515-1524. doi: 10.3892/ijmm.2016.2748 |
|  | Alox5 | Arachidonic acid metabolism, Ca^2+^, cAMP and lipid signaling, NF-κB signaling, innate immune system | immune cell lines | h | Busch S, Auth E, Scholl F, Huenecke S, Koehl U, Suess B, Steinhilber D. 2015. 5-lipoxygenase is a direct target of miR-19a-3p and miR-125b-5p. J Immunol. 194(4):1646-53. doi: 10.4049/jimmunol.1402163 |
| **miR-21-5p** | PDCD4 | MAPK signaling, Akt signaling, mTOR signaling | different immune cell types | h.m | reviewed in Sheedy F. Turning 21: Induction of miR-21 as a Key Switch in the Inflammatory Response. Front Immunol. 2015;6:19. doi: 10.3389/fimmu.2015.00019 |
|  | PTEN | AKT signaling, mTOR signaling, IL-2 pathway, CD28 signaling in T-helper cell |  |  |  |
|  | IL12p35 | IL-12 family signaling, MAPK signaling, TGF-β/Smad Signaling, DC developmental lineage pathway, T helper cell type 1 differentiation, TLR signaling |  |  |  |
|  | CXCL10 | STAT3 signaling, MAPK signaling, IL-6 signaling, TLR signaling, RIG-I/MDA5 mediated induction of IFN type 1 pathways, Rho family GTPases signaling, NF-κB signaling, cytokine signaling in immune system |  |  |  |
|  | Ppara | PPAR signaling, cAMP signaling, MAPK signaling |  |  |  |
| **miR-22-3p** | Sp1 | PARP signaling, TGF-β/Smad signaling | HCC | h | Chen J, Wu FX, Luo HL, Liu JJ, Luo T, Bai T, Li LQ, Fan XH. Berberine upregulates miR-22-3p to suppress hepatocellular carcinoma cell proliferation by targeting Sp1. Am J Transl Res. 2016;8(11):4932-4941 |
|  | Tcf7 | canonical and non-canonical Wnt signaling | HCC cell line |  | Kaur K, Vig S, Srivastava R, Mishra A, Singh VP, Srivastava AK, Datta M. Elevated Hepatic miR-22-3p Expression Impairs Gluconeogenesis by Silencing the Wnt-Responsive Transcription Factor Tcf7. Diabetes. 2015;64(11):3659-69. doi: 10.2337/db14-1924 |

| **miRNA** | **Target mRNA** | **Affected pathway^1^** | **Cell type^2^** | **Species^3^** | **Reference** |
| --- | --- | --- | --- | --- | --- |
| **miR-23b-3p** | Tgif1 | TGF-β/Smad Signaling | differentiating keratinocytes | h | Barbollat-Boutrand L, Joly-Tonetti N, Dos Santos M, Metral E, Boher A, Masse I, Berthier-Vergnes O, Bertolino P, Damour O, Lamartine J. MicroRNA-23b-3p regulates human keratinocyte differentiation through repression of TGIF1 and activation of the TGF-ß-SMAD2 signalling pathway. Exp Dermatol. 2017;26(1):51-57. doi: 10.1111/exd.13119 |
|  | Pten | AKT signaling, mTOR signaling, IL-2 pathway, CD28 signaling in T helper cell | RCC cell lines | h | Zaman MS, Thamminana S, Shahryari V, Chiyomaru T, Deng G, Saini S, Majid S, Fukuhara S, Chang I, Arora S, Hirata H, Ueno K, Singh K, Tanaka Y, Dahiya R. Inhibition of PTEN gene expression by oncogenic miR-23b-3p in renal cancer. PLoS One. 2012; 7(11):e50203. doi: 10.1371/journal.pone.0050203 |
| **miR-24-3p** | CopS5 | HIF signaling, TGF-β/Smad Signaling | NPC | h | Wang S, Pan Y Zhang R, Xu T, Wu W, Zhang R, Wang C, Huang H, Calin CA, Yang H, Claret FX. Hsa-miR-24-3p increases nasopharyngeal carcinoma radiosensitivity by targeting both the 3'UTR and 5'UTR of Jab1/CSN5. Oncogene. 2016;35(47):6096-6108. doi: 10.1038/onc.2016.147 |
|  | Fgf11 | Akt signaling, RAS signaling, MAPK signaling, TGF-β/Smad signaling, JAK/STAT signaling, PPAR signaling, Rho family GTPases, NFAT in immune response | NPS cell lines, sera of NPC patients | h | Ye SB, Zhang H, Cai TT, Liu YN, Ni JJ, He J, Peng JY, Chen QY, Mo HY, Jun-Cui, Zhang XS, Zeng YX, Li J. Exosomal miR-24-3p impedes T-cell function by targeting FGF11 and serves as a potential prognostic biomarker for nasopharyngeal carcinoma. J Pathol. 2016;240(3):329-340. doi: 10.1002/path.4781 |
|  | HNF4A | MAPK signaling, HIF signaling, AMPK signaling, TGFß/Smad signaling | HCC cell line | h | Salloum-Asfar S, Arroyo AB, Teruel-Montoya R, García-Barberá N, Roldán V, Vicente V, Martínez C, González-Conejero R. MiRNA-Based Regulation of Hemostatic Factors through Hepatic Nuclear Factor-4 Alpha. PLoS One. 2016;11(5):e0154751. doi: 10.1371/journal.pone.0154751 |

| **miRNA** | **Target mRNA** | **Affected pathway^1^** | **Cell type^2^** | **Species^3^** | **Reference** |
| --- | --- | --- | --- | --- | --- |
| **miR-24-3p** | Prdx-6 | innate immune system | GCA | h | Li Q, Wang N, Wei H, Li C, Wu J, Yang G. miR-24-3p Regulates Progression of Gastric Mucosal Lesions and Suppresses Proliferation and Invasiveness of N87 via Peroxiredoxin 6. Dig Dis Sci. 2016;61(12):3486-3497 |
|  | Trim11 | Class I MHC mediated antigen processing and presentation, innate immune system | colon cancer | h | Yin Y, Zhong J, Li SW, Li JZ, Zhou M, Chen Y, Sang Y, Liu L. TRIM11, a direct target of miR-24-3p, promotes cell proliferation and inhibits apoptosis in colon cancer. Oncotarget. 2016;7(52):86755-86765. doi: 10.18632/oncotarget.13550 |
| **miR-26b-5p** | Traf5 | MAPK signaling, NF-κB signaling, CD40 signaling, Apoptosis and survival anti-apoptotic TNF-α/NF-κB/Bcl-2 pathway | melanoma cells | h,m | Li M, Long C, Yang G, Luo Y, Du H. MiR-26b inhibits melanoma cell proliferation and enhances apoptosis by suppressing TRAF5-mediated MAPK activation. Biochem Biophys Res Commun. 2016;471(3):361-7. doi: 10.1016/j.bbrc.2016.02.021 |
|  | Map3k7 | TLR signaling, NOD receptor signaling, MAPK signaling, NF-κB signaling, Wnt signaling, immune response IL-1 signaling pathway, Adaptive immune system | HCC cell lines | h | Zhao N, Wang R, Zhou L, Zhu Y, Gong J, Zhuang SM. MicroRNA-26b suppresses the NF-κB signaling and enhances the chemosensitivity of hepatocellular carcinoma cells by targeting TAK1 and TAB3. Mol Cancer. 2014;13:35. doi: 10.1186/1476-4598-13-35 |
|  | Tab3 | TLR signaling, NOD receptor signaling, MAPK signaling, NF-κB signaling, WNT signaling, IL-1 signaling, TNF-α signaling, cytokine signaling in immune system |  |  |  |
| **miR-27a-3p** | Mapk10 | TLR signaling, NOD receptor signaling, MAPK signaling, AKT signaling, inflammasome activation pathways, IL-1 and L-2 signaling, CD40 signaling | NPC | h | Li L, Luo Z. Dysregulated miR-27a-3p promotes nasopharyngeal carcinoma cell proliferation and migration by targeting Mapk10. Oncol Rep. 2017;37(5):2679-2687. doi: 10.3892/or.2017.5544 |
|  | YAP1 | PARP signaling, AKT signaling, WNT signaling, TGF-ß/Smad signaling | CSCC | h | Zeng G, Xun W, Wei K, Yang Y, Shen H. MicroRNA-27a-3p regulates epithelial to mesenchymal transition via targeting YAP1 in oral squamous cell carcinoma cells. Oncol Rep. 2016;36(3):1475-82. doi: 10.3892/or.2016.4916 |

| **miRNA** | **Target mRNA** | **Affected pathway^1^** | **Cell type^2^** | **Species^3^** | **Reference** |
| --- | --- | --- | --- | --- | --- |
| **miR-27a-3p** | Fbxw7 | NOTCH signaling, Class I MHC mediated antigen processing and presentation | ESCC | h | Wu XZ, Wang KP, Song HJ, Xia JH, Jiang Y, Wang YL. MiR-27a-3p promotes esophageal cancer cell proliferation via F-box and WD repeat domain-containing 7 (FBXW7) suppression. Int J Clin Exp Med. 2015;8(9):15556-62 |
|  | Wnt3a | canonical and non-canonical WNT signaling, mTOR signaling | melanocytes | m | Zhao Y, Wang P, Meng J, Ji Y, Xu D, Chen T, Fan R, Yu X, Yao J, Dong C. MicroRNA-27a-3p Inhibits Melanogenesis in Mouse Skin Melanocytes by Targeting Wnt3a. Int J Mol Sci. 2015;16(5):10921-33. doi: 10.3390/ijms160510921 |
| **miR-27b-3p** | Creb1 | cAMP signaling, TLR signaling, MAPK signaling, AKT signaling, AMPK signaling, TGF-ß/Smad signaling | BC | h | Zhu J, Zou Z, Nie P, Kou X, Wu B, Wang S, Song Z, He J. Downregulation of microRNA-27b-3p enhances tamoxifen resistance in breast cancer by increasing NR5A2 and CREB1 expression. Cell Death Dis. 2016;7(11):e2454. doi: 10.1038/cddis.2016.361 |
| **miR-30a-5p** | IL6R, IL6ST | TGF-ß/Smad signaling, MAPK family pathway, AKT signaling, JAK/STAT signaling | induced regulatory T cells | h | Schiavinato JLDS, Haddad R, Saldanha-Araujo F, Baiochi J, Araujo AG, Santos Scheucher P, Covas DT, Zago MA, Panepucci RA. TGF-beta/atRA-induced Tregs express a selected set of microRNAs involved in the repression of transcripts related to Th17 differentiation. Sci Rep. 2017;7(1):3627. doi: 10.1038/s41598-017-03456-8 |
|  | Zeb2 | TGF-ß/Smad signaling | RCC | h | Chen Z, Zhang J, Zhang Z, Feng Z, Wei J, Lu J, Fang Y, Liang Y, Cen J, Pan Y, Huang Y, Zhou F, Chen W, Luo J. The putative tumor suppressor microRNA-30a-5p modulates clear cell renal cell carcinoma aggressiveness through repression of ZEB2. Cell Death Dis. 2017;8(6):e2859. doi: 10.1038/cddis.2017.252 |
|  | Snai1 | WNT signaling, TGF-ß/SMD signaling | diabetic cataracts | h | Zhang L, Wang Y, Li W, Tsonis PA, Li Z, Xie L, Huang Y. MicroRNA-30a Regulation of Epithelial-Mesenchymal Transition in Diabetic Cataracts Through Targeting SNAI1. Sci Rep. 2017;7(1):1117. doi: 10.1038/s41598-017-01320-3 |
| **miR-30a-5p** | CD73 (NT5E) | adenosine nucleotides degradation, HIF signaling, NF-κB signaling | NSCL | h | Zhu J, Zeng Y, Li W, Qin H, Lei Z, Shen D, Gu D, Huang JA, Liu Z. CD73/NT5E is a target of miR-30a-5p and plays an important role in the pathogenesis of non-small cell lung cancer. Mol Cancer. 2017;16(1):34. doi: 10.1186/s12943-017-0591-1 |
|  | PIK3R2 | AKT signaling, AMPK Signaling, actin dynamics signaling, TLR signaling | NSCL cell lines | h | Meng F, Wang F, Wang L, Wong SC, Cho WC, Chan LW. MiR-30a-5p Overexpression May Overcome EGFR-Inhibitor Resistance through Regulating PI3K/AKT Signaling Pathway in Non-small Cell Lung Cancer Cell Lines. Front Genet. 2016;7:197. |
|  | Itgb3 | MAPK signaling, integrin signaling, actin dynamics signaling, AKT signaling | CRC | h | Wei W, Yang Y, Cai J, Cui K, Li RX, Wang H, Shang X, Wei D. MiR-30a-5p Suppresses Tumor Metastasis of Human Colorectal Cancer by Targeting ITGB3.Cell Physiol Biochem. 2016;39(3):1165-76. doi: 10.1159/000447823 |
|  | Ube3c | Class I MHC mediated antigen processing and presentation, TGF-ß/Smad Signaling | BC cell lines | h | Xiong J, Wei B, Ye Q, Liu W. MiR-30a-5p/UBE3C axis regulates breast cancer cell proliferation and migration. Biochem Biophys Res Commun. 2016;pii: S0006-291X(16)30381-3. doi: 10.1016/j.bbrc.2016.03.069 |
|  | Aeg-1 | cytoskeleton remodeling regulation of actin cytoskeleton by Rho GTPases | HCC | h | He R, Yang L, Lin X, Chen X, Lin X, Wei F, Liang X, Luo Y, Wu Y, Gan T, Dang Y, Chen G. MiR-30a-5p suppresses cell growth and enhances apoptosis of hepatocellular carcinoma cells via targeting AEG-1. Int J Clin Exp Pathol. 2015;8(12):15632-41 |
|  | Mtdh | AKT signaling | HCC cell lines | h | Li WF, Dai H, Ou Q, Zuo GQ, Liu CA. Overexpression of microRNA-30a-5p inhibits liver cancer cell proliferation and induces apoptosis by targeting MTDH/PTEN/AKT pathway. Tumour Biol. 2016;37(5):5885-95. doi: 10.1007/s13277-015-4456-1 |

| **miRNA** | **Target mRNA** | **Affected pathway^1^** | **Cell type^2^** | **Species^3^** | **Reference** |
| --- | --- | --- | --- | --- | --- |
| **miR-30a-5p** | Runx2 | DC developmental lineage pathway, NOTCH signaling | giant cell tumor of bone | h | Huang Q, Jiang Z, Meng T, Yin H, Wang J, Wan W, Cheng M, Yan W, Liu T, Song D, Chen H, Wu Z, Xu W, Li Z, Zhou W, Xiao J. MiR-30a inhibits osteolysis by targeting RunX2 in giant cell tumor of bone. Biochem Biophys Res Commun. 2014;453(1):160-5. doi: 10.1016/j.bbrc.2014.09.076 |
|  | Sept7 | MAPK signaling, Rho GTPase signaling | GBM cell lines | h | Jia Z, Wang K, Wang G, Zhang A, Pu P. MiR-30a-5p antisense oligonucleotide suppresses glioma cell growth by targeting SEPT7. PLoS One. 2013;8(1):e55008. doi: 10.1371/journal.pone.0055008 |
|  | Prdm1 | NF-κB signaling, WNT signaling | glioma | h | Wang X, Wang K, Han L, Zhang A, Shi Z, Zhang K, Zhang H, Yang S, Pu P, Shen C, Yu C, Kang C. PRDM1 is directly targeted by miR-30a-5p and modulates the Wnt/β-catenin pathway in a Dkk1-dependent manner during glioma growth. Cancer Lett. 2013;331(2):211-9. doi: 10.1016/j.canlet.2013.01.005 |
|  | CD99 | integrin signaling, transendothelial migration of leukocytes | Ewing tumor | h | Franzetti GA, Laud-Duval K, Bellanger D, Stern MH, Sastre-Garau X, Delattre O. MiR-30a-5p connects EWS-FLI1 and CD99, two major therapeutic targets in Ewing tumor. Oncogene. 2013;32(33):3915-21 |
| **miR-30c-5p** | Socs3 | Class I MHC mediated antigen processing and presentation, interferon gamma signaling, IL-10 signaling, IL-23 signaling | BC cell line | h | Yen MC, Shih YC, Hsu YL, Lin ES, Lin YS, Tsai EM, Ho YW, Hou MF, Kuo PL. Isolinderalactone enhances the inhibition of SOCS3 on STAT3 activity by decreasing miR-30c in breast cancer. Oncol Rep. 2016;35(3):1356-64. doi: 10.3892/or.2015.4503 |
| **miR-30e-5p** | ITGA6 | MAPK pathway, integrin pathway | CRC | h | Laudato S, Patil N, Abba ML, Leupold JH, Benner A, Gaiser T, Marx A, Allgayer H. P53-induced miR-30e-5p inhibits colorectal cancer invasion and metastasis by targeting ITGA6 and ITGB1. Int J Cancer. 2017;141(9):1879-1890.doi: 10.1002/ijc.30854 |
|  | ITGB1 | MAPK pathway, AKT pathway, integrin pathway |  |  |  |

| **miRNA** | **Target mRNA** | **Affected pathway^1^** | **Cell type^2^** | **Species^3^** | **Reference** |
| --- | --- | --- | --- | --- | --- |
| **miR-31-5p** | Tollip | TLR signaling, NF-κB signaling, IL-1 signaling pathway, cytokine signaling in immune system, innate immune system | intestinal epithelial cells | m | Sugi Y, Takahashi K, Kurihara K, Nakata K, Narabayashi H, Hamamoto Y, Suzuki M, Tsuda M, Hanazawa S, Hosono A, Kaminogawa S. Post-Transcriptional Regulation of Toll-Interacting Protein in the Intestinal Epithelium. PLoS One. 2016;11(10):e0164858. doi: 10.1371/journal.pone.0164858 |
|  | NFAT | RANKL/RANK Signaling, cAMP signaling, PKC signaling, NF-κB signaling, canonical and non-canonical WNT signaling, T cell differentiation | CD4^+^ T cells | h | Fan W, Liang D, Tang Y, Qu B, Cui H, Luo X, Huang X, Chen S, Higgs BW, Jallal B, Yao Y, Harley JB, Shen N. Identification of microRNA-31 as a novel regulator contributing to impaired interleukin-2 production in T cells from patients with systemic lupus erythematosus. Arthritis Rheum. 2012;64(11):3715-25. doi: 10.1002/art.34596 |
|  | RhoA | Rho GTPase signaling, actin dynamics signaling, canonical and non-canonical WNT signaling, blood-brain barrier and immune cell transmigration: ICAM-1/CD54 signaling |  |  |  |
| **miR-93-5** | Pten | AKT signaling, mTOR Signaling, IL-2 pathway, CD28 signaling in T helper cell | cardiomyocytes | h | Ke ZP, Xu P, Shi Y, Gao AM. MicroRNA-93 inhibits ischemia-reperfusion induced cardiomyocyte apoptosis by targeting PTEN. Oncotarget. 2016;7(20):28796-805. doi: 10.18632/oncotarget.8941 |
|  | HIF-1a | HIF signaling, NF-κB signaling, MAPK signaling | HCC cells | h | Jiang Y, Zhu Y, Wang X, Gong J, Hu C, Guo B, Zhu B, Li Y. Temporal regulation of HIF-1 and NF-κB in hypoxic hepatocarcinoma cells. Oncotarget. 2015;6(11):9409-19. |
|  | RhoC | Rho GTPase signaling, actin dynamics signaling | OC | h | Chen X, Chen S, Xiu YL, Sun KX, Zong ZH, Zhao Y. RhoC is a major target of microRNA-93-5P in epithelial ovarian carcinoma tumorigenesis and progression. Mol Cancer. 2015;14:31. doi: 10.1186/s12943-015-0304-6 |
|  | Pten | AKT signaling, mTOR signaling, IL-2 pathway, CD28 signaling in T helper cell | HCC cells | h | Ohta K, Hoshino H, Wang J, Ono S, Iida Y, Hata K, Huang SK, Colquhoun S, Hoon DS. MicroRNA-93 activates c-Met/PI3K/Akt pathway activity in hepatocellular carcinoma by directly inhibiting PTEN and CDKN1A. Oncotarget. 2015;6(5):3211-24 |

| **miRNA** | **Target mRNA** | **Affected pathway^1^** | **Cell type^2^** | **Species^3^** | **Reference** |
| --- | --- | --- | --- | --- | --- |
| **miR-126-3p** | Pik3r2 | AKT signaling, actin dynamics signaling, AMPK signaling, MAPK signaling, mTOR signaling, common cytokine receptor gamma-chain family signaling | synovial fibroblasts  (rheumatoid arthritis) | h | Qu Y, Wu J, Deng JX, Zhang YP, Liang WY, Jiang ZL, Yu QH, Li J. MicroRNA-126 affects rheumatoid arthritis synovial fibroblast proliferation and apoptosis by targeting PIK3R2 and regulating PI3K-AKT signal pathway. Oncotarget. 2016;7(45):74217-74226. doi: 10.18632/oncotarget.12487 |
|  | Cxcr4 | AKT signaling, JAK/STAT signaling, NF-κB signaling, chemokine signaling | CRC cell lines | h | Liu Y, Zhou Y, Feng X, An P, Quan X, Wang H, Ye S, Yu C, He Y, Luo H. MicroRNA-126 functions as a tumor suppressor in colorectal cancer cells by targeting CXCR4 via the AKT and ERK1/2 signaling pathways. Int J Oncol. 2014;44(1):203-10. doi: 10.3892/ijo.2013.2168 |
|  | Kras | MAPK signaling | GBM | h | Li Y, Li Y, Ge P, Ma C. MiR-126 Regulates the ERK Pathway via Targeting KRAS to Inhibit the Glioma Cell Proliferation and Invasion. Mol Neurobiol. 2017;54(1):137-145. doi: 10.1007/s12035-015-9654-8 |
| **miR-135a-5p** | Foxo1 | AKT signaling, MAPK signaling, common cytokine receptor gamma-chain family signaling | HCC cell lines | h | Shi H, Fang R, Li Y, Li L, Zhang W, Wang H, Chen F, Zhang S, Zhang X, Ye L. The oncoprotein HBXIP suppresses gluconeogenesis through modulating PCK1 to enhance the growth of hepatoma cells. Cancer Lett. 2016;382(2):147-156. doi: 10.1016/j.canlet.2016.08.025 |
| **miR-139-5p** | Cxcr4 | AKT signaling, JAK/STAT signaling, NF-κB signaling, chemokine signaling | GC | h | Bao W, Fu HJ, Xie QS, Wang L, Zhang R, Guo ZY, Zhao J, Meng YL, Ren XL, Wang T, Li Q, Jin BQ, Yao LB, Wang RA, Fan DM, Chen SY, Jia LT, Yang AG. HER2 interacts with CD44 to up-regulate CXCR4 via epigenetic silencing of microRNA-139 in gastric cancer cells. Gastroenterology. 2011;141(6):2076-2087.e6. doi: 10.1053/j.gastro.2011.08.050. |
|  | IRS1 | AKT signaling, common cytokine receptor gamma-chain family signaling pathways | 3T3-L1 cell line | h | Mi L, Chen Y, Zheng X, Li Y, Zhang Q, Mo D, Yang G. 2015. MicroRNA-139-5p Suppresses 3T3-L1 Preadipocyte Differentiation Through Notch and IRS1/PI3K/Akt Insulin Signaling Pathways. J Cell Biochem. 2015;116(7):1195-204. doi: 10.1002/jcb.25065 |
|  | Notch1 | NOTCH signaling, T cell differentiation |  |  |  |

| **miRNA** | **Target mRNA** | **Affected pathway^1^** | **Cell type^2^** | **Species^3^** | **Reference** |
| --- | --- | --- | --- | --- | --- |
| **miR-139-5p** | Nfkb1 | TLR signaling, TNF-α signaling, NF-κB signaling, MAPK signaling | BC | h | Krishnan K, Steptoe AL, Martin HC, Pattabiraman DR, Nones K, Waddell N, Mariasegaram M, Simpson PT, Lakhani SR, Vlassov A, Grimmond SM, Cloonan N. miR-139-5p is a regulator of metastatic pathways in breast cancer. RNA. 2013;19(12):1767-80. doi: 10.1261/rna.042143.113 |
|  | Rap1b | MAPK signaling, integrin signaling, cytoskeletal signaling | primary tissues of miR-139^-/-^ mice | m | Zou F, Mao R, Yang L, Lin S, Lei K, Zheng Y, Ding Y, Zhang P, Cai G, Liang X, Liu J. Targeted deletion of miR-139-5p activates MAPK, NF-κB and STAT3 signaling and promotes intestinal inflammation and colorectal cancer. FEBS J. 2016;283(8):1438-52. doi: 10.1111/febs.13678 |
|  | Tcf-4 | canonical and non-canonical WNT signaling |  |  | Gu W, Li XM, Wang J. miR-139 regulates theproliferation and invasion of hepatocellular carcinomathrough the WNT/TCF-4 pathway. Oncol Rep 2014;31(1):397–404. doi: 10.3892/or.2013.2831 |
| **miR-140-3p** | ATP8A1 | innate immune system | NSLC | h | Dong W, Yao C, Teng X, Chai J, Yang X, Li B. MiR-140-3p suppressed cell growth and invasion by downregulating the expression of ATP8A1 in non-small cell lung cancer. Tumour Biol. 2016;37(3):2973-85. doi: 10.1007/s13277-015-3452-9 |
|  | Rala | Ras signaling | MSC differentiation | h | Karlsen TA1, Jakobsen RB, Mikkelsen TS, Brinchmann JE. microRNA-140 targets RALA and regulates chondrogenic differentiation of human mesenchymal stem cells by translational enhancement of SOX9 and ACAN. Stem Cells Dev. 2014;23(3):290-304. doi: 10.1089/scd.2013.0209 |
|  | CD38 | Ca^2+^ signaling | ASMC | h | Jude JA, Dileepan M, Subramanian S, Solway J, Panettieri RA Jr, Walseth TF, Kannan MS. miR-140-3p regulation of TNF-α-induced CD38 expression in human airway smooth muscle cells. Am J Physiol Lung Cell Mol Physiol. 2012;303(5):L460-8. doi: 10.1152/ajplung.00041.2012 |

| **miRNA** | **Target mRNA** | **Affected pathway^1^** | **Cell type^2^** | **Species^3^** | **Reference** |
| --- | --- | --- | --- | --- | --- |
| **miR-140-5p** | Stat1 | JAK/STAT signaling, NF-κB signaling, MAPK signaling, TLR signaling, common cytokine receptor gamma-chain family signaling pathways | PBMC | h | Guan H, Singh UP, Rao R, Mrelashvili D, Sen S, Hao H, Zumbrun EE, Singh NP, Nagarkatti PS, Nagarkatti M. Inverse correlation of expression of microRNA-140-5p with progression of multiple sclerosis and differentiation of encephalitogenic T helper type 1 cells. Immunology. 2016;147(4):488-98. doi: 10.1111/imm.12583 |
| **miR-148a-3p** | Cckbr | Ca^2+^ signaling | GC | h | Cao H, Liu Z, Wang R, Zhang X, Yi W, Nie G, Yu Y, Wang G, Zhu M. miR-148a suppresses human renal cell carcinoma malignancy by targeting AKT2. Oncol Rep. 2016;37(1):147-154. doi: 10.3892/or.2016.5257 |
|  | Pten | AKT signaling, mTOR signaling, IL-2 pathway, CD28 signaling in T helper cell | renal tissues  (lupus nephritis) | h | Qingjuan L, Xiaojuan F, Wei Z, Chao W, Pengpeng K, Hongbo L, Sanbing Z, Jun H, Min Y, Shuxia L. 2016. miR-148a-3p overexpression contributes to glomerular cell proliferation by targeting PTEN in lupus nephritis. Am J Physiol Cell Physiol. 2015;310(6):C470-8. doi: 10.1152/ajpcell.00129.2015 |
|  | Map3k4 | MAPK signaling, TNF-α signaling | CSCC cells | h | Luo Q, Li W, Zhao T, Tian X, Liu Y, Zhang X. Role of miR-148a in cutaneous squamous cell carcinoma by repression of MAPK pathway. Arch Biochem Biophys. 2015;583:47-54. doi: 10.1016/j.abb.2015.07.022 |
|  | Map3k9 | MAPK signaling, IL2 pathway |  |  |  |
|  | Bach2 | NF-κB signaling | B cells | h,m | Porstner M, Winkelmann R, Daum P, Schmid J, Pracht K, Côrte-Real J, Schreiber S, Haftmann C, Brandl A, Mashreghi MF, Gelse K, Hauke M, Wirries I, Zwick M, Roth E, Radbruch A, Wittmann J, Jäck HM. miR-148a promotes plasma cell differentiation and targets the germinal center transcription factors Mitf and Bach2. Eur J Immunol. 2015;45(4):1206-15. doi: 10.1002/eji.201444637 |
|  | Mitf | RANKL/RANK signaling, MAPK signaling, canonical and non-canonical WNT signaling |  |  |  |

| **miRNA** | **Target mRNA** | **Affected pathway^1^** | **Cell type^2^** | **Species^3^** | **Reference** |
| --- | --- | --- | --- | --- | --- |
| **miR-148-3p** | Iκbkb | TLR signaling, MAPK signaling, TNF-α signaling, NF-κB signaling, AKT signaling, IL-1 signaling, Class I MHC mediated antigen processing and presentation | aortic valve interstitial cells | h | Patel V, Carrion K1, Hollands A, Hinton A, Gallegos T, Dyo J, Sasik R, Leire E, Hardiman G, Mohamed SA, Nigam S, King CC, Nizet V, Nigam V. The stretch responsive microRNA miR-148a-3p is a novel repressor of IKBKB, NF-κB signaling, and inflammatory gene expression in human aortic valve cells. FASEB J. 2015;29(5):1859-68. doi: 10.1096/fj.14-257808 |
|  | relA | TLR signaling, NOD receptor signaling, NF-κB signaling, adaptive immune system | cardiac muscle | h | Bao JL, Lin L. MiR-155 and miR-148a reduce cardiac injury by inhibiting NF-κB pathway during acute viral myocarditis. Eur Rev Med Pharmacol Sci. 2014;18(16):2349-56. |
| **miR-150-5p** | Akt3 | AKT signaling, mTOR signaling, AMPK signaling, JAK/STAT signaling, common cytokine receptor gamma-chain family signaling pathways | CD4^+^ T cells | h | Sang W, Sun C, Zhang C, Zhang D, Wang Y, Xu L, Zhang Z, Wei X, Pan B, Yan D, Zhu F, Yan Z, Cao J, Loughran TP Jr, Xu K. MicroRNA-150 negatively regulates the function of CD4(+) T cells through AKT3/Bim signaling pathway. Cell Immunol. 2016;306-307:35-40. doi: 10.1016/j.cellimm.2016.05.007 |
|  | ARRB2 | MAPK signaling, canonical and non-canonical WNT signaling, hedgehog signaling, NOTCH signaling | CD4^+^ T cells | h | Sang W, Wang Y, Zhang C, Zhang D, Sun C, Niu M, Zhang Z, Wei X, Pan B, Chen W, Yan D, Zeng L, Loughran TP Jr, Xu K. MiR-150 impairs inflammatory cytokine production by targeting ARRB-2 after blocking CD28/B7 costimulatory pathway. Immunol Lett. 2016;172:1-10. doi: 10.1016/j.imlet.2015.11.001 |
|  | Stat1 | JAK/STAT signaling, NF-κB signaling, MAPK signaling, TLR signaling, common cytokine receptor gamma-chain family signaling pathways | CD4^+^ T cell lines | h | Moles R, Bellon M, Nicot C. STAT1: A Novel Target of miR-150 and miR-223 Is Involved in the Proliferation of HTLV-I-Transformed and ATL Cells. Neoplasia 2015;17(5):449-62. doi: 10.1016/j.neo.2015.04.005 |
| **miR-188-5p** | LAPTM4B | AKT signaling, lysosome | prostate cancer | h | Zhang H, Qi S, Zhang T, Wang A, Liu R, Guo J, Wang Y, Xu Y. miR-188-5p inhibits tumour growth and metastasis in prostate cancer by repressing LAPTM4B expression. Oncotarget. 2015;6(8):6092-104 |
| **miR-208a-3p** | Pdcd4 | AKT signaling, mTOR signaling, MAPK signaling, Interferon type I signaling pathways | GC | h | Yin K, Liu M, Zhang M, Wang F, Fen M, Liu Z, Yuan Y, Gao S, Yang L, Zhang W, Zhang J, Guo B, Xu J, Liang H, Chen X, Guan W. miR-208a-3p suppresses cell apoptosis by targeting PDCD4 in gastric cancer. Oncotarget. 2016;7(41):67321-67332. doi: 10.18632/oncotarget.12006 |
| **miR-211-5p** | Setbp1 | AKT signaling, TGF-β/Smadsignaling | BC | h | Chen LL, Zhang ZJ, Yi ZB, Li JJ. 2017. MicroRNA-211-5p suppresses tumour cell proliferation, invasion, migration and metastasis in triple-negative breast cancer by directly targeting SETBP1. Br J Cancer. 2017;117(1):78-88. doi: 10.1038/bjc.2017.150 |
|  | Sox11 | MAPK signaling | TC | h | Wang L, Shen YF, Shi ZM, Shang XJ, Jin DL, Xi F. Overexpression miR-211-5p hinders the proliferation, migration, and invasion of thyroid tumor cells by downregulating SOX11. J Clin Lab Anal. 2017. doi: 10.1002/jcla.22293 |
|  | Zeb2 | TGF-ß/Smad signaling | HCC | h | Jiang G, Wen L, Deng W, Jian Z, Zheng H. Regulatory role of miR-211-5p in hepatocellular carcinoma metastasis by targeting ZEB2. Biomed Pharmacother. 2017;90:806-812. doi: 10.1016/j.biopha.2017.03.081 |
|  | Snai1 | WNT signaling | RCC | h | Wang K, Jin W, Jin P, Fei X, Wang X, Chen X. miR-211-5p Suppresses Metastatic Behavior by Targeting SNAI1 in Renal Cancer. Mol Cancer Res. 2017;15(4):448-456. doi: 10.1158/1541-7786.MCR-16-0288 |
|  | Stat1 | JAK/STAT signaling, NF-κB signaling, MAPK signaling, TLR signaling, common cytokine receptor gamma-chain family signaling pathways | CD4^+^ T cell lines | h | Moles R, Bellon M, Nicot C. STAT1: A Novel Target of miR-150 and miR-223 Is Involved in the Proliferation of HTLV-I-Transformed and ATL Cells. Neoplasia. 2015;17(5):449-62. doi: 10.1016/j.neo.2015.04.005 |
| **miR-338-3p** | Adam17 | NOTCH signaling, TNF-α signaling, degradation of extracellular matrix | GC cell lines | h | Chen JT, Yao KH, Hua L, Zhang LP, Wang CY, Zhang JJ. MiR-338-3p inhibits the proliferation and migration of gastric cancer cells by targeting ADAM17. Int J Clin Exp Pathol. 2015;8(9):10922-8 |

| **miRNA** | **Target mRNA** | **Affected pathway^1^** | **Cell type^2^** | **Species^3^** | **Reference** |
| --- | --- | --- | --- | --- | --- |
| **miR-338-3p** | Akt3 | AKT signaling, mTOR signaling, AMPK signaling, JAK/STAT signaling, common cytokine receptor gamma-chain family signaling pathways | TC | m | Sui GQ, Fei D, Guo F, Zhen X, Luo Q, Yin S, Wang H. MicroRNA-338-3p inhibits thyroid cancer progression through targeting AKT3. Am J Cancer Res. 2017;7(5):1177-1187 |
|  | Hif-1a | HIF signaling, NF-κB signaling, MAPK signaling | NPC | h | Shan Y, Li X, You B, Shi S, Zhang Q, You Y. MicroRNA-338 inhibits migration and proliferation by targeting hypoxia-induced factor 1α in nasopharyngeal carcinoma. Oncol Rep. 2015;34(4):1943-52. doi: 10.3892/or.2015.4195 |
|  | PREX2a | AKT signaling, regulation of RAC1 activity | sebocytes | h | Liu J, Cao L, Feng Y, Li Y, Li T. MiR-338-3p inhibits TNF-α-induced lipogenesis in human sebocytes. Biotechnol Lett. 2017 Jun 9. doi: 10.1007/s10529-017-2369-3 |
|  | Rab14 | AMPK signaling pathway, GTPase signaling | NSCL | h | Sun J, Feng X, Gao S, Xiao Z. microRNA-338-3p functions as a tumor suppressor in human non‑small‑cell lung carcinoma and targets Ras-related protein 14. Mol Med Rep. 2015;11(2):1400-6. doi: 10.3892/mmr.2014.2880 |
|  | Rankl | AKT signaling, MAPK signaling, NF-κB signaling | osteoclasts | h | Zhang XH, Geng GL, Su B, Liang CP, Wang F, Bao JC. MicroRNA-338-3p inhibits glucocorticoid-induced osteoclast formation through RANKL targeting. Genet Mol Res. 2016;15(3). doi: 10.4238/gmr.15037674 |
|  | Runx2 | DC developmental lineage pathway, NOTCH signaling | osteoblasts | m | Liu H, Sun Q, Wan C, Li L, Zhang L, Chen Z. MicroRNA-338-3p regulates osteogenic differentiation of mouse bone marrow stromal stem cells by targeting Runx2 and Fgfr2. J Cell Physiol. 2014229(10):1494-502. doi: 10.1002/jcp.24591 |
|  | Sox4 | MAPK signaling, WNT signaling | NSCL cell lines | h | Li Y, Chen P, Zu L, Liu B, Wang M, Zhou Q. MicroRNA-338-3p suppresses metastasis of lung cancer cells by targeting the EMT regulator Sox4. Am J Cancer Res. 2016;15;6(2):127-40 |

| **miRNA** | **Target mRNA** | **Affected pathway^1^** | **Cell type^2^** | **Species^3^** | **Reference** |
| --- | --- | --- | --- | --- | --- |
| **miR-338-3p** | SphK2 | Ca^2+^ signaling, Sphingolipid metabolism, FcγR-mediated phagocytosis | NSCL cell lines | h | Zhang G, Zheng H, Zhang G, Cheng R, Lu C, Guo Y, Zhao G. MicroRNA-338-3p suppresses cell proliferation and induces apoptosis of non-small-cell lung cancer by targeting sphingosine kinase 2. Cancer Cell Int. 2017;17:46. doi: 10.1186/s12935-017-0415-9 |
|  | Zeb2 | TGF-β/Smad Signaling | GC cell lines | h | Huang N, Wu Z, Lin L, Zhou M, Wang L, Ma H, Xia J, Bin J, Liao Y, Liao W. MiR-338-3p inhibits epithelial-mesenchymal transition in gastric cancer cells by targeting ZEB2 and MACC1/Met/Akt signaling. Oncotarget. 2015;20;6(17):15222-34 |
| **miR-340-5p** | Mitf | RANKL/RANK signaling, MAPK signaling, canonical and non-canonical WNT signaling | melanoma | h | Poenitzsch Strong AM, Setaluri V, Spiegelman VS. MicroRNA-340 as a modulator of RAS-RAF-MAPK signaling in melanoma. Arch Biochem Biophys. 2014;563:118-24. doi: 10.1016/j.abb.2014.07.012 |
|  | Nras | Ras signaling, MAPK signaling, cAMP signaling, AKT signaling | GBM | h | Fiore D, Donnarumma E, Roscigno G, Iaboni M, Russo V, Affinito A, Adamo A, De Martino F, Quintavalle C, Romano G, Greco A, Soini Y, Brunetti A, Croce CM, Condorelli G. miR-340 predicts glioblastoma survival and modulates key cancer hallmarks through down-regulation of NRAS. Oncotarget. 2015;7(15):19531-47. doi: 10.18632/oncotarget.6968 |
|  | Stat3 | JAK/STAT signaling, common cytokine receptor gamma-chain family signaling pathways | HCC | h | Xiong Q, Wu S, Wang J, Zeng X, Chen J, Wei M, Guan H, Fan C, Chen L, Guo D, Sun G. 2017. Hepatitis B virus promotes cancer cell migration by downregulating miR-340-5p expression to induce STAT3 overexpression. Cell Biosci. 7:16. doi: 10.1186/s13578-017-0144-8 |
| **miR-362-5p** | PI3K-C2ß | AKT signaling, MAPK signaling | neuroblastoma | h | Wu K, Yang L, Chen J, Zhao H, Wang J, Xu S, Huang Z. miR-362-5p inhibits proliferation and migration of neuroblastoma cells by targeting phosphatidylinositol 3-kinase-C2β. FEBS Lett. 2015;589(15):1911-9 |

| **miRNA** | **Target mRNA** | **Affected pathway^1^** | **Cell type^2^** | **Species^3^** | **Reference** |
| --- | --- | --- | --- | --- | --- |
| **miR-365-3p** | Kcnh2 | Rac signaling | neurons | m | Pan Z, Zhang M, Ma T, Xue ZY, Li GF, Hao LY, Zhu LJ, Li YQ, Ding HL, Cao JL. 2016. Hydroxymethylation of microRNA-365-3p Regulates Nociceptive Behaviors via Kcnh2. J Neurosci. 36(9):2769-81. doi: 10.1523/JNEUROSCI.3474-15.2016 |
| **miR-451a** | Akt1 | AKT signaling, common cytokine receptor gamma-chain family signaling pathways | TC | h | Minna E, Romeo P, Dugo M, De Cecco L, Todoerti K, Pilotti S, Perrone F, Seregni E, Agnelli L, Neri A, Greco A, Borrello MG. miR-451a is underexpressed and targets AKT/mTOR pathway in papillary thyroid carcinoma. Oncotarget. 2016;7(11):12731-47. doi: 10.18632/oncotarget.7262 |
|  | Mif | innate immune system |  |  |  |
| **miR-483-3p** | Smad4 | TGF-β/Smad Signaling | GC | h | Hao J, Zhang S, Zhou Y, Hu X, Shao C. 2010. MicroRNA 483-3p suppresses the expression of DPC4/Smad4 in pancreatic cancer. FEBS Lett. 2011 Jan 3;585(1):207-13. doi: 10.1016/j.febslet.2010.11.039 |
|  | PRKC | PKC signaling | OC | h | Arrighetti N, Cossa G, De Cecco L, Stucchi S, Carenini N, Corna E, Gandellini P, Zaffaroni N, Perego P, Gatti L. PKC-alpha modulation by miR-483-3p in platinum-resistant ovarian carcinoma cells. Toxicol Appl Pharmacol. 2016;1;310:9-19. doi: 10.1016/j.taap.2016.08.005 |
|  | CTNNB1 | WNT signaling, blood-brain barrier and immune cell transmigration | adenocarcinoma | h | Veronese A, Visone R, Consiglio J, Acunzo M, Lupini L, Kim T, Ferracin M, Lovat F, Miotto E, Balatti V, D'Abundo L, Gramantieri L, Bolondi L, Pekarsky Y, Perrotti D, Negrini M, Croce CM. Mutated beta-catenin evades a microRNA-dependent regulatory loop. Proc Natl Acad Sci U S A. 2011;108(12):4840-5. doi: 10.1073/pnas.1101734108 |
| **miR-483-5p** | RhoA | Rho GTPse signaling, actin dynamics signaling, canonical and non-canonical WNT signaling, blood-brain barrier and immune cell transmigration: ICAM-1/CD54 signaling | adipose tissue  (multiple symmetric lipomatosis) | h | Chen K, He H, Xie Y, Zhao L, Zhao S, Wan X, Yang W, Mo Z. miR-125a-3p and miR-483-5p promote adipogenesis via suppressing the RhoA/ROCK1/ERK1/2 pathway in multiple symmetric lipomatosis. Sci Rep. 2015;5:11909. doi: 10.1038/srep11909 |
| **miR-483-5p** | Erk1 | MAPK signaling |  |  |  |
|  | RhoGDI1 | Rho GTPase signaling, cytoskeletal signaling | lung adenocarcinoma | h | Song Q, Xu Y, Yang C, Chen Z, Jia C, Chen J, Zhang Y, Lai P, Fan X, Zhou X, Lin J, Li M, Ma W, Luo S, Bai X. miR-483-5p promotes invasion and metastasis of lung adenocarcinoma by targeting RhoGDI1 and ALCAM. Cancer Res. 2014;74(11):3031-42. doi: 10.1158/0008-5472.CAN-13-2193. |
| **miR-494-3p** | Socs6 | JAK/STAT signaling | hematopoietic stem/progenitor cells | h | Rontauroli S, Norfo R, Pennucci V, Zini R, Ruberti S, Bianchi E, Salati S, Prudente Z, Rossi C, Rosti V, Guglielmelli P, Barosi G, Vannucchi A, Tagliafico E, Manfredini R. miR-494-3p overexpression promotes megakaryocytopoiesis in primary myelofibrosis hematopoietic stem/progenitor cells by targeting SOCS6. Oncotarget. 2017;8(13):21380-21397. doi: 10.18632/oncotarget.15226 |
| **miR-574-5p** | Foxn3 | TLR9 signaling | lung cancer cell lines | h | Li Q, Li X, Guo Z, Xu F, Xia J, Liu Z, Ren T. MicroRNA-574-5p was pivotal for TLR9 signaling enhanced tumor progression via down-regulating checkpoint suppressor 1 in human lung cancer. PLoS One. 2012;7(11):e48278. doi: 10.1371/journal.pone.0048278 |
|  | Qki | MAPK signalling, WNT signaling | CRC | h | Ji S, Ye G, Zhang J, Wang L, Wang T, Wang Z, Zhang T, Wang G, Guo Z, Luo Y, Cai J, Yang JY. miR-574-5p negatively regulates Qki6/7 to impact β-catenin/Wnt signalling and the development of colorectal cancer. Gut. 2013;62(5):716-26. doi: 10.1136/gutjnl-2011-301083 |
| **miR-709** | GSK-3β | WNT signaling | macrophage cell line | h | Li M, Chen H, Chen L, Chen Y, Liu X, Mo D. miR-709 modulates LPS-induced inflammatory response through targeting GSK-3β. Int Immunopharmacol. 2016;36:333-8. doi: 10.1016/j.intimp.2016.04.005 |

| **miRNA** | **Target mRNA** | **Affected pathway^1^** | **Cell type^2^** | **Species^3^** | **Reference** |
| --- | --- | --- | --- | --- | --- |
| **miR-721** | PPAR-γ | PPAR signaling, AMPK signaling | dipocyte cell line | h | Ke B, Ke X, Wan X, Yang Y, Huang Y, Qin J, Hu C, Shi L. Astragalus polysaccharides attenuates TNF-α-induced insulin resistance via suppression of miR-721 and activation of PPAR-γ and PI3K/AKT in 3T3-L1 adipocytes. Am J Transl Res. 2017;9(5):2195-2206 |
| **miR-1187** | Casp8 | Apoptosis, TNFR1 pathway, TLR signaling | hepatic cells (acute liver failure model) | m | Yu DS, An FM, Gong BD, Xiang XG, Lin LY, Wang H, Xie Q. The regulatory role of microRNA-1187 in TNF-α-mediated hepatocyte apoptosis in acute liver failure. Int J Mol Med. 2012;29(4):663-8. doi: 10.3892/ijmm.2012.888 |

**^1^**  functions of miRNA target mRNAs retrieved from GeneCard database, section `Pathways & Interactions´ (http://www.genecards.org/).

**^2^** ASMC, airway smooth muscle cells; BC, breast cancer; CRC, colorectal cancer; CSCC, cutaneous squamous cell carcinoma; ESCC, esophageal squamous cell carcinomas; GBM, glioblastoma; GC, gastric cancer; HCC, hepatocellular carcinoma; MSC, mesenchymal stem cell; NPC, nasopharyngeal carcinoma; NSCL, non-small-cell lung cancer; OC, ovarian cancer; PBMC, peripheral blood mononuclear cells; RCC, renal cell carcinoma; TC, thyroid cancer.

**^3^** h, human; m: mouse.


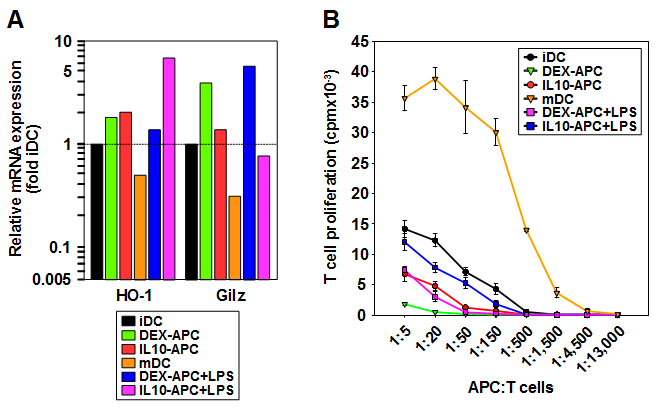


**Figure S1. Immuno-phenotype of differentially tolerized APC populations. (A)** Expression levels of HO-1 and Gilz mRNA by the different APC populations at unstimulated state and after stimulation with LPS was monitored by QPCR, and normalized to the corresponding iDC expression level. Data denote the mean expression levels of duplicates. Graphs are representative for 2 independent experiments. **(B)** The allo T cell stimulatory capacity of the different APC populations at either state of activation was monitored as described in the materials and methods section. Data denote the mean±SEM of triplicates. **(A,B)** Results are representative of 2 independent experiments.


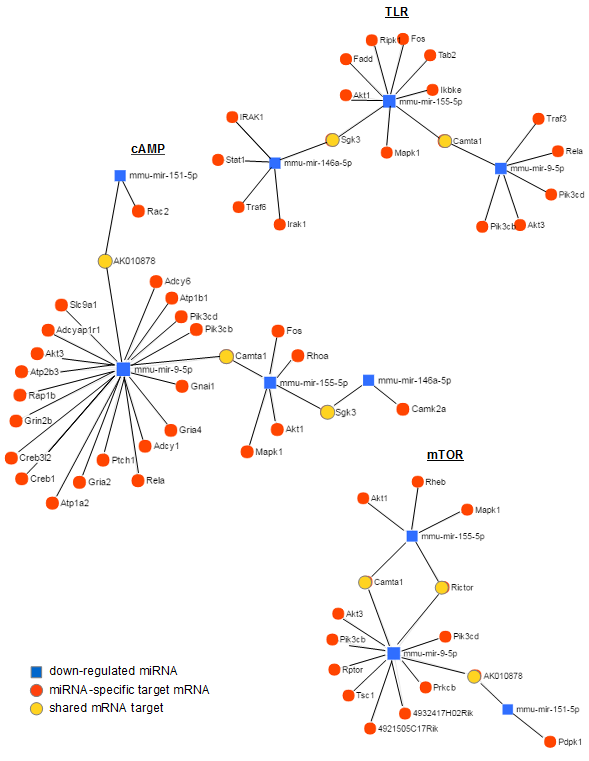


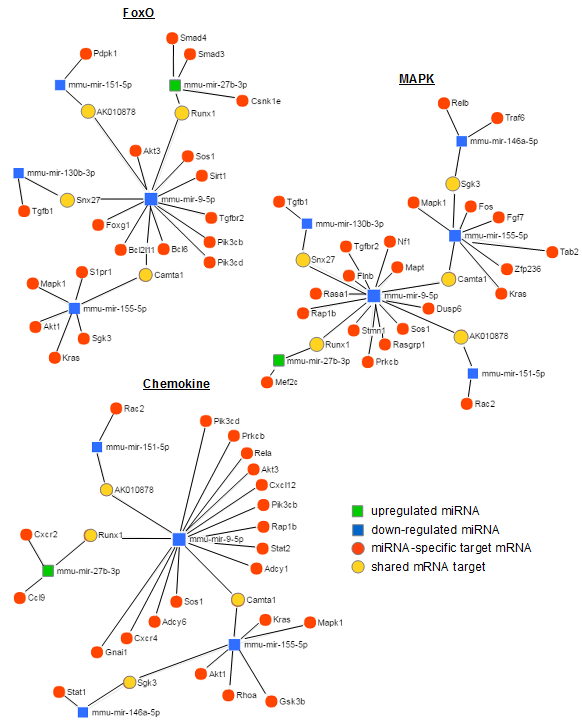


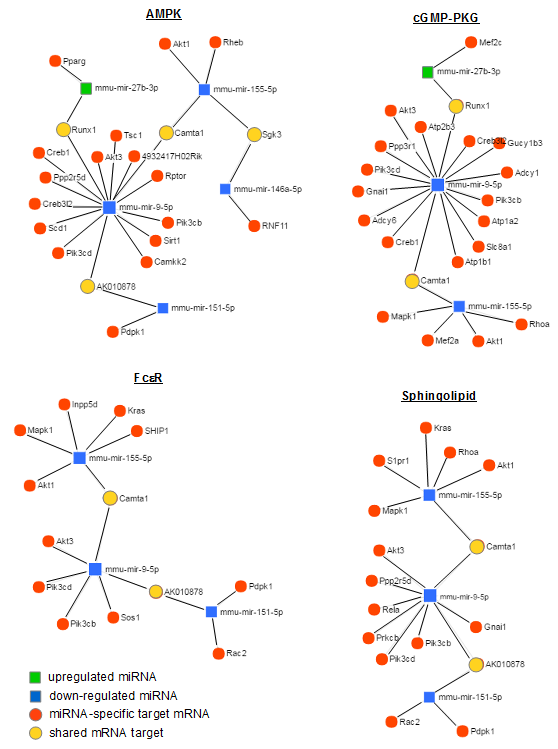


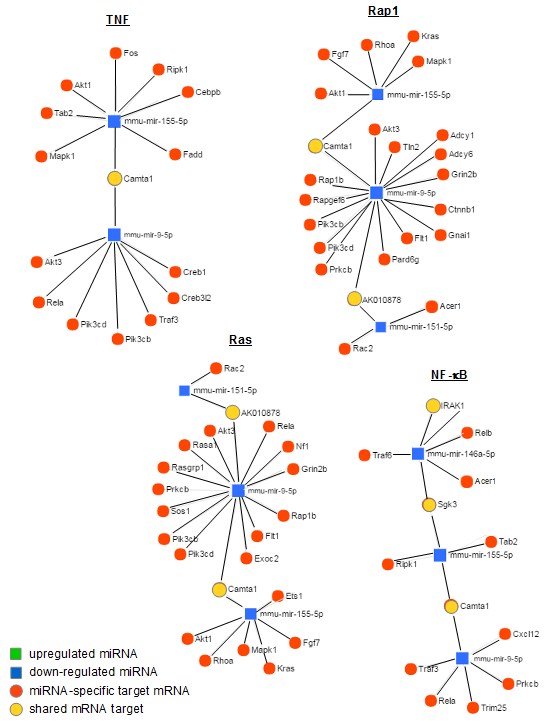


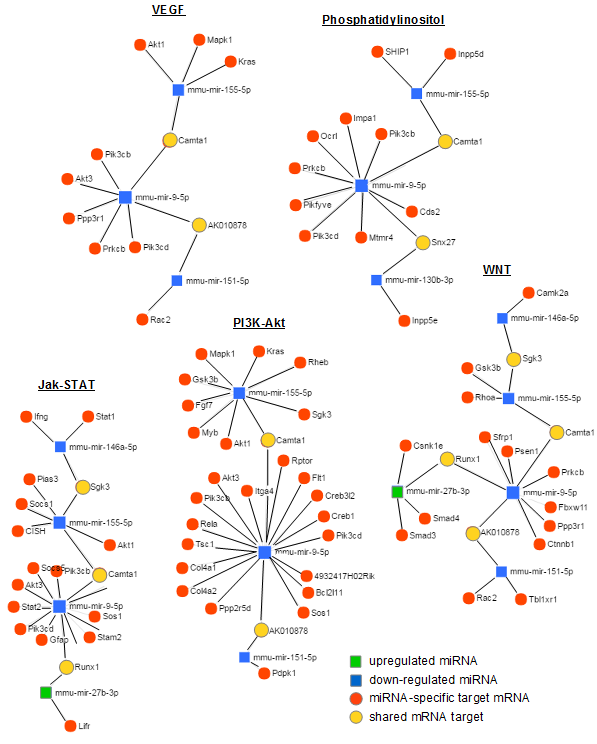


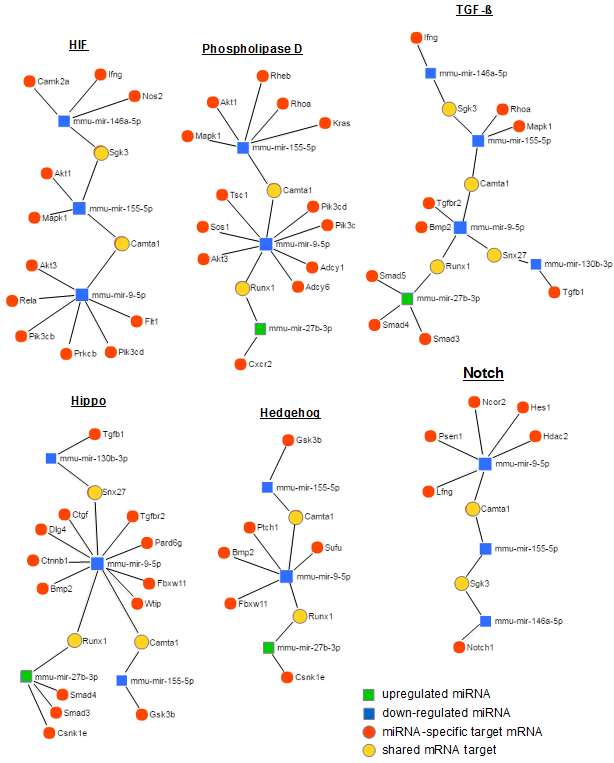


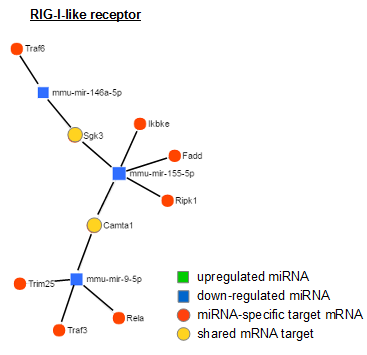


**Fig. S2: Predicted miRNA-mRNA target interaction networks within immune cell-relevant pathways, based on miRNA species convergingly regulated in tolerogenic DEX-APCs and IL10-APCs at unstimulated state as compared with iDCs.** Schemes show interaction networks related to signal transduction. Qualitative changes in miRNA expression in tolerogenic APCs versus iDCs are indicated. mRNA targets of a single or several miRNAs are labeled.

**Figure S3:** **Transfection rate of iDCs transfected with siRNA.** iDCs were transfected with a FITC-labeled control siRNA (see materials and methods section). One day after the 2nd transfection, BMDCs were harvested. FITC expression in iDCs left untreated (grey line) and transfected with FITC-siRNA (black line) was monitored by flow cytometry (representative of 5 independent experiments).


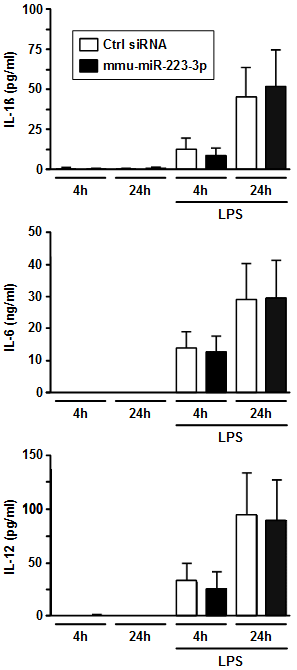


**Figure S4. BMDCs transfected with mmu-miR-223-3p mimick express cytokines at unaltered extent.** C57BL/6 BMDCs were transfected two times in parallel with mmu-miR-223-3p mimick and scrambled control siRNA, respectively (see materials and methods section). After 3h, aliquots were stimulated overnight with LPS (1 µg/ml). Supernatants of differentially transfected BMDCs were harvested at 4h and 24h after the 2nd round of transfection, and cytokine levels were assessed by ELISA as described (*Bros M, Jährling F, Renzing A, Wiechmann N, Dang NA, Sutter A, Ross R, Knop J, Sudowe S, Reske-Kunz AB. 2007. A newly established murine immature dendritic cell line can be differentiated into a mature state, but exerts tolerogenic function upon maturation in the presence of glucocorticoid. Blood 109:3820-9*). Data denote the mean±SEM of 4 independent experiments.

**Figure S5:** **BMDCs engineered to overexpress mmu-miR-223-3p fail to acquire stimulation-induced CD4^+^ T cell stimulatory capacity.** One day after the second transfection of C57BL/6 BMDCs with mmu-miR-223-3p mimick and scrambled control siRNA, respectively (see materials and methods section), transfectants and control BMDCs (iDC, mDC) cultured in parallel were treated with ovalbumin (1 µg/ml; Sigma-Aldrich, Deisenhofen, Germany). After 3h, aliquots were stimulated overnight with LPS (1 µg/ml). On the next day, BMDCs were harvested and thoroughly washed. Splenic ovalbumin peptide-specific CD4^+^ OT-II T cells were labeled with CFSE (10 µM; Thermo Fisher, Waltham, MA). BMDCs (10^5^) were cocultured with OT-II T cells (5x10^5^) in a volume of 2 ml in 12 well plates (Greiner Bio-One) for 3d. Then, cultures were incubated with antibodies specific for CD3 (clone: 145-2C11, fluorochrome: PE-Cy5) and CD4 (GK1.5, APC), and samples were assayed by flow cytometry. Histograms show CFSE intensities of CD3^+^CD4^+^ T cells, and are representative of 2 independent experiments. Frequencies of T cells that have undergone mitosis are denoted.
